# Adaptive path tracking control of unmanned agricultural machinery with fixed-time super-twisting sliding mode based on RLS-ELM

**DOI:** 10.3389/fpls.2025.1678648

**Published:** 2025-09-19

**Authors:** Zhijian Chen, Jianjun Yin, Sheikh Muhammad Farhan, Zhenhua Lin, Maile Zhou

**Affiliations:** ^1^ School of Agricultural Engineering, Jiangsu University, Zhenjiang, China; ^2^ School of Mechanical Engineering, Tongji University, Shanghai, China

**Keywords:** unmanned agricultural machines, path tracking, terminal sliding mode, super-twisting sliding mode control, extreme learning machine

## Abstract

**Introduction:**

Accurate path tracking is essential for achieving intelligent operation in unmanned agricultural machinery. To address the limitations of traditional agricultural machine path tracking methods, which are susceptible to high frequency oscillations and external disturbances, this study proposes a fixed time super-twisting sliding mode adaptive path-tracking control for unmanned agricultural vehicles.

**Methods:**

The approach utilizes a Regularized Least Squares Extreme Learning Machine (RLS-ELM) to improve robustness and adaptability under certain operating conditions. A generalized terminal sliding mode surface is first designed by incorporating both lateral and heading deviations of the vehicle. Next, a Super-Twisting Sliding Mode control law is developed to perform path tracking, while the RLS-ELM is used to estimate and compensate for unknown disturbances. The stability of the proposed control system is verified through the construction of a new Lyapunov function.

**Results:**

The control algorithm is validated via field experiments on an agricultural platform. Results show that, compared to the Fixed-Time Generalized Terminal Super Twisting control method (FGST) and the Fixed-Time Sliding Mode Controller (FTSMC), the Extreme Learning Machine-Adaptive Fixed-Time Generalized Super-Twisting (ELM-AFGST) method reduces lateral mean absolute errors by 24.5% and 27.4%, respectively, and decreases heading mean absolute errors by 5.4% and 30.8%, respectively. These findings demonstrate that the proposed path tracking method provides a solid theoretical framework for high-precision path tracking of unmanned agricultural machines.

## 1 Introduction

At present, the advancement of intelligent, automated, and information-driven agricultural machinery has become an inevitable trend and is widely used in various agricultural production practices (Jin et al., 2021; Zhang et al., 2024). Among these technologies, autonomous navigation technology for agricultural machinery is the core technology that enables the realization of unmanned farms, playing a crucial role in the entire process of plowing, planting, crop management, and harvesting. Its implementation significantly enhances operational efficiency, improves task quality, optimizes land utilization, and reduces both operator fatigue and the risk of agricultural accidents (Bai et al., 2023). In addition, autonomous navigation technology also provides more possibilities for the development of agricultural robots, further promoting agricultural modernization and laying an important foundation for the future development of agricultural technology (Yang et al., 2022). Autonomous navigation technology is mainly composed of three key components: environment perception, path planning, and path tracking (Rovira-Más et al., 2020; Wang et al., 2024). Path tracking plays a critical role in modern agriculture automation by enabling vehicles to follow pre-defined trajectories with high precision. This functionally reduces unnecessary turns and repeated operations, thus enhancing operational efficiency and productivity. In contrast, manual driving is prone to human error, which can compromise the precision of field tasks and lead to increased operator fatigue. Automated path tracking not only improves operational consistency but also enables agricultural vehicles to better adapt to diverse and irregular terrain conditions (Sun et al., 2024). However, real-world agricultural environments, such as muddy fields, uneven surfaces, and rough roads, pose significant challenges to stable and reliable path tracking (Si et al., 2019). Therefore, improving the robustness and accuracy of path tracking systems under these conditions is essential for advancing the practical deployment of autonomous agricultural machinery (Chen et al., 2023; Cui et al., 2024).

When operating in outdoor environments, unmanned agricultural machines rely on a real-time dynamic positioning system to obtain accurate positional data for effective path tracking (Xie et al., 2023). To enhance the path-tracking performance of unmanned agricultural machines equipped with navigation systems, researchers have proposed various control strategies (Carpio et al., 2020). These models usually employ lateral and heading deviation as state variables, and have been widely used in path tracking and control of unmanned agricultural vehicles due to their practicality and effectiveness (Luo et al., 2022). To achieve accurate path tracking control of unmanned agricultural machines, various control strategies have been employed in the development of path tracking systems. These strategies are mainly categorized into model-free and model-based control algorithms. Model-free control algorithms include Proportional-Integral-Derivative (PID) control, neural network control, and fuzzy logic control (Cheng and Lu, 2018). Model free control does not require an exact model, but typically relies on heuristic rules, empirical parameters, or historical data. In complex scenarios, control strategies lacking model guidance may be difficult to adapt quickly and require manual intervention or long-term online learning adjustments. Model-based control algorithms primarily include model predictive control (MPC), pure tracking control, and Sliding Mode Control (SMC), among others. However, MPC requires a balance between model accuracy, computational efficiency, and parameter tuning (Xu et al., 2021). Still, pure tracking control forward view distance needs to be manually adjusted according to the speed and the curvature of the path, and it cannot be adaptive (Ma et al., 2022). Compared to the above control strategies, SMC is widely used in engineering due to its strong robustness and adaptability to system parameter uncertainties and external disturbances, enabling it to deal with uncertain situations effectively (Ding et al., 2023).

It is worth noting that due to the advantages of the above SMC has a certain degree of application in engineering practice, but the actual system, the high frequency switching of the control signal leads to the system state in the sliding surface near the chattering, and ultimately affects the stability of the driving of agricultural machinery, how to reduce the chattering and at the same time to maintain the system’s interference resistance is still a difficult problem that needs to be solved (Zhang et al., 2022). Therefore, to overcome the limitations of existing Siding Mode Control methods, particularly the high-frequency jitter and parameter sensitivity issues, many scholars have adopted this approach to enhance system stability. (Ding et al., 2023). introduced a path tracking model in the presence of unknown matched and unmatched disturbances and designed a second-order perturbation observer combined with an adaptive SMC strategy based on the barrier function. The adverse effects of matched and unmatched disturbances are counteracted, while the jitter problem of the designed controller is attenuated. Ultimately, simulations and experiments verify the superiority of the designed controller. Super-Twisting Sliding Mode Control (STSMC) is an improved algorithm for second order SMC. By introducing the second order sliding mode structure, the switching process of the control inputs is smoothed, and the high-frequency jitter phenomenon in traditional SMC is effectively reduced, while maintaining strong robustness. For example, (Yang et al., 2024). proposed a FSTSM control algorithm with additional linear and concomitant terms to enhance path tracking accuracy and robustness for the tracking control problem of unmanned agricultural machines under unknown matching disturbances. (Ji et al., 2024). inspired by the traditional STSMC, this study introduces perturbation observation and feedforward compensation, constructs a composite STSMC strategy, and applies it to the pre-aim error dynamic model path tracking control, and verifies the effectiveness of the algorithm through the vehicle dynamic simulation tool and hardware-in-the-loop simulation platform.

Furthermore, when applying kinetic or kinematic tracking models, it is often assumed in the above literature that the upper bound of the system perturbation is known; however, in real operations, the perturbation is random and usually not directly accessible through sensors. Considering the physical limitations of real scenarios and equipment, it can only be ensured that the disturbances are bounded, but their exact upper bound is often difficult to determine. Therefore, it is crucial to develop a SMC method that can effectively handle disturbances, even when their maximum value is unknown. The structure of this article’s sections is as follows: In Section 2, the hardware composition and software architecture of the experimental platform are established, the workflow of the path tracking system is explained, and a vehicle kinematic model is developed to lay the foundation for subsequent controller design; In Section 3, a generalized terminal sliding surface is designed and a super-twisting sliding mode control law is constructed to achieve convergence control of path tracking errors, while an RLS-ELM network is introduced to estimate and compensate for unknown disturbances, and a Lyapunov function is built to verify system stability; In Section 4, through simulation experiments, the proposed method is compared with various existing controllers, and lateral error and heading error under different parameters are analyzed, while the disturbance estimation accuracy of RLS-ELM versus other observers is compared, and field tests are conducted to verify the superiority of Extreme Learning Machine-Adaptive Fixed-Time Generalized Super-Twisting (ELM-AFGST) in real environments; In Section 5, conclusions and prospects are provided, summarizing the advantages of the proposed ELM-AFGST control strategy, pointing out the limitations of the current model, and indicating future optimization directions. The main contributions of this study are as follows:

- This article proposes an adaptive mechanism based on the RLS-ELM framework, which can achieve effective control without precise knowledge of the disturbance upper bound. By recursively updating the model output weights in real-time, the dependence on prior knowledge of disturbances is reduced, solving the problem of random disturbances in actual agricultural operations that are difficult to obtain directly through sensors.
- Using hyper spiral sliding mode control as the core strategy, a fixed time generalized terminal sliding mode surface is designed to overcome the limitation of traditional disturbance observers that cannot be directly embedded into the control law. The controller can effectively constrain the trajectory of the combine harvester under external disturbances, and the convergence of the system is theoretically verified through Lyapunov stability analysis, improving the robustness of path tracking.
- The effectiveness of the ELM-AFGST controller was verified through simulation testing and field testing of a combine harvester chassis equipped with RTK-GNSS. The experimental results confirm that it can achieve high-precision path tracking, providing a solution that combines theoretical support and practical value for the development of autonomous navigation technology for agricultural machinery.

## 2 Experimental materials and system modeling

### 2.1 Structure of the automatic navigation system for unmanned agricultural machines

#### 2.1.1 Agricultural machinery, automatic navigation platform

**Figure 1:**
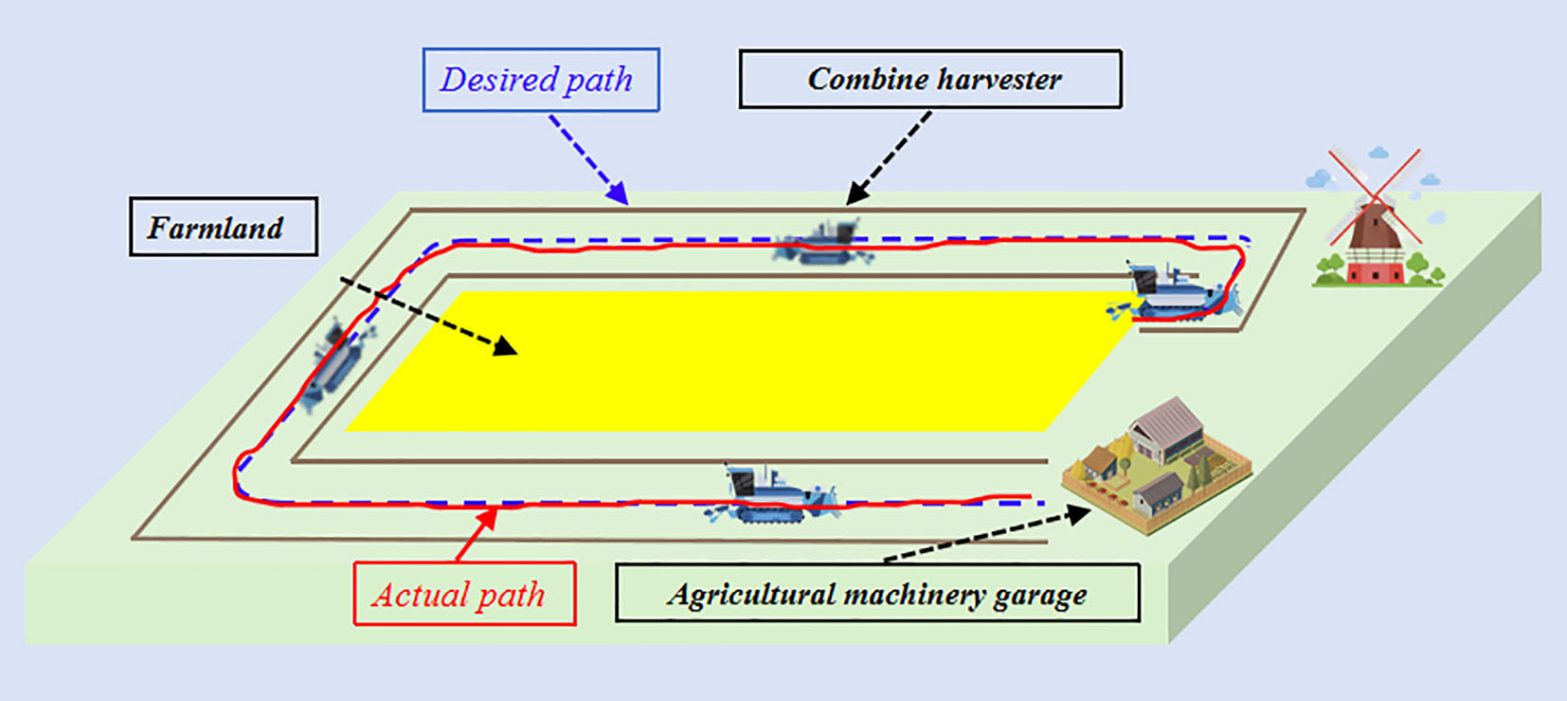
Scenarios of common turnaround driving operations of combine harvester.

As a key technology in precision agriculture, the unmanned agricultural machinery automatic navigation system enables the autonomous operation of the entire agricultural equipment process through multimodal data fusion and intelligent control technology, thus reducing labor costs. When the path tracking performance of the unmanned agricultural machinery automatic navigation system is insufficient, it will affect the efficiency of agricultural machinery operation, so it is necessary to design an autonomous navigation system to achieve the transfer control of agricultural equipment in the field after the completion of the task. To verify the effectiveness of the proposed algorithm research, this study implements a path tracking control scheme on a combine harvester platform as shown in **Figure 1**. This study considers a common driving operation scenario of a harvester travelling, i.e., the reference path often consists of straight lines and curves, which are often used in agricultural driving scenarios.

In this study, the world 4LZK-5.0FQ tracked harvester is used as the research object, which is equipped with a hydraulic continuously variable speed (HST) system and a paired tracked travelling mechanism to enable it to adapt to a variety of complex terrains, such as mud, ruggedness, wetlands, etc., without terrain limitations. The diesel engine-based tracked vehicle has a powerful powertrain with high power, stable performance, and low cost. Therefore, it can efficiently complete a variety of farmland operations, and it is significant to realize the automatic navigation and travel capabilities of this type of vehicle. The main technical parameters of the vehicle are shown in **Table 1**.

**Table 1:** Main technical parameters of world 4LZK-5.0FQ crawler harvester.

Technical Parameter	Value	Performance of walking system	Value
L×W×H/mm	5830×3450×3020	Transmission type	World dedicated 110 gearbox
Overall quality/kg	4760	Theoretical speed/km·h^-1^	0.9-6.5
Cutting table width/mm	2360	Track gauge/mm	1250
Feed rate/kg·s^-1^	5	Track pitch(mm)×Number×W(mm)	90×63×500

#### 2.1.2 Hardware components of the autonomous navigation system

**Figure 2:**
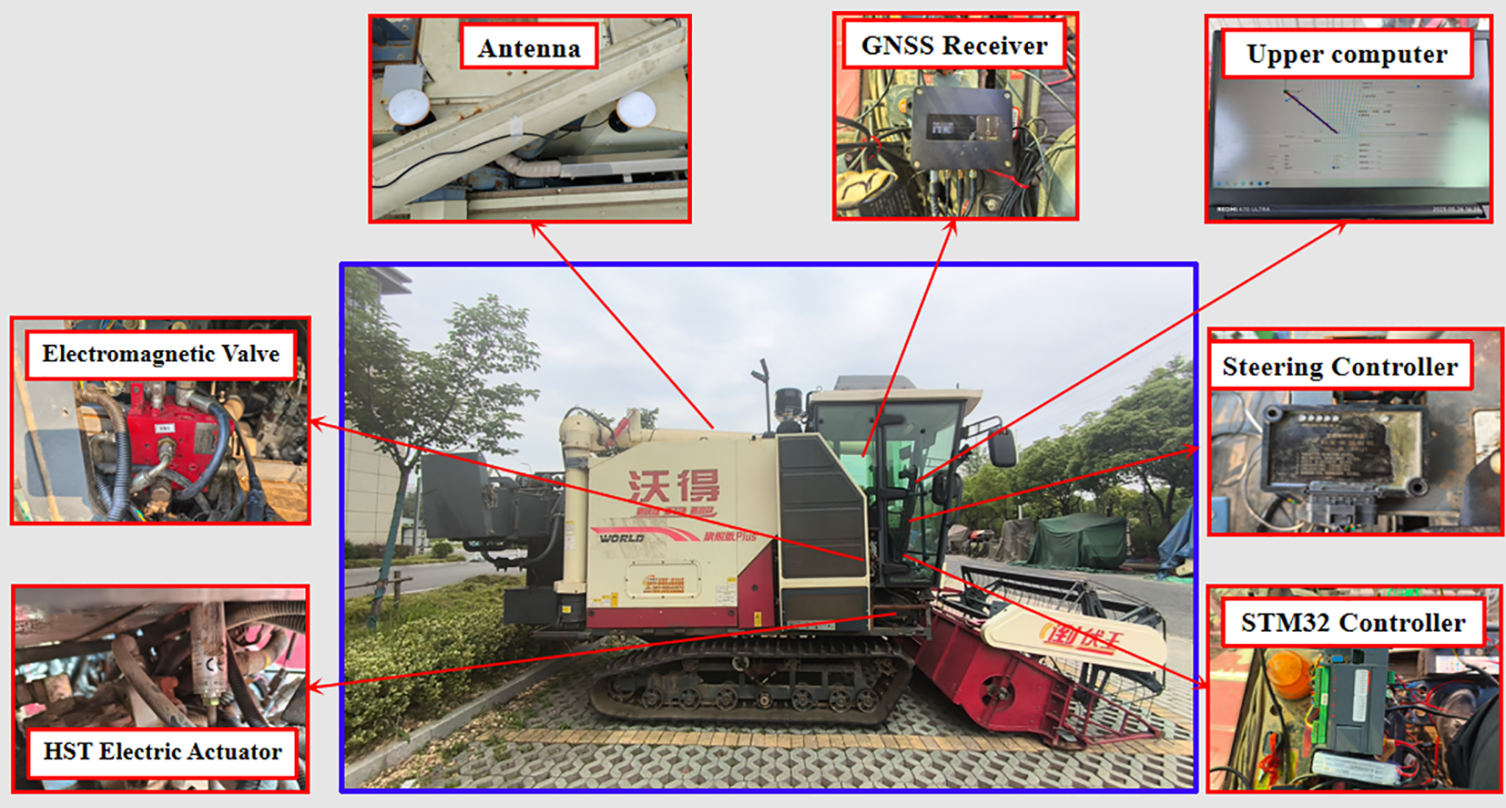
Overall structure of the combine harvester system.

The harvester autonomous navigation hardware system is mainly composed of an external environment sensing module, a control decision-making module, and an underlying driver module. The external environment sensing module includes RTK-GNSS for positioning (Thousand Seek Navigation, G200mini), which adopts dual-antenna working mode and receives real-time information such as latitude, longitude and heading angle when the harvester is moving, as well as the rotation speed of the active track wheel (E6B2-CWZ5B encoder, OMRON, Japan), which is used to obtain the real-time speed of the vehicle. The control decision-making module (Lenovo laptop computer with CPU frequency of 3.6 GHz and memory of 16GB) receives the information transferred from the sensing module and the underlying driving module. The CPU main frequency is 3.6 GHz with 16GB memory (flow), accepts the information transmitted from the sensing module, carries out the vehicle position information processing and data saving and other operations, runs the path tracking control algorithm on the STM32F429 lower computer, carries out the real-time motion control of the vehicle, the controller accepts the commands issued by the upper computer through the CAN bus, and finally sends the control commands to the controller and accepts the commands from the upper computer through the CAN bus, and finally sends the control commands to the aforementioned modified bottom drive module, to control the harvester and realize the vehicle walking and steering movements, the overall system composition of the modified combine harvester is shown in **Figure 2**.

**Figure 3:**
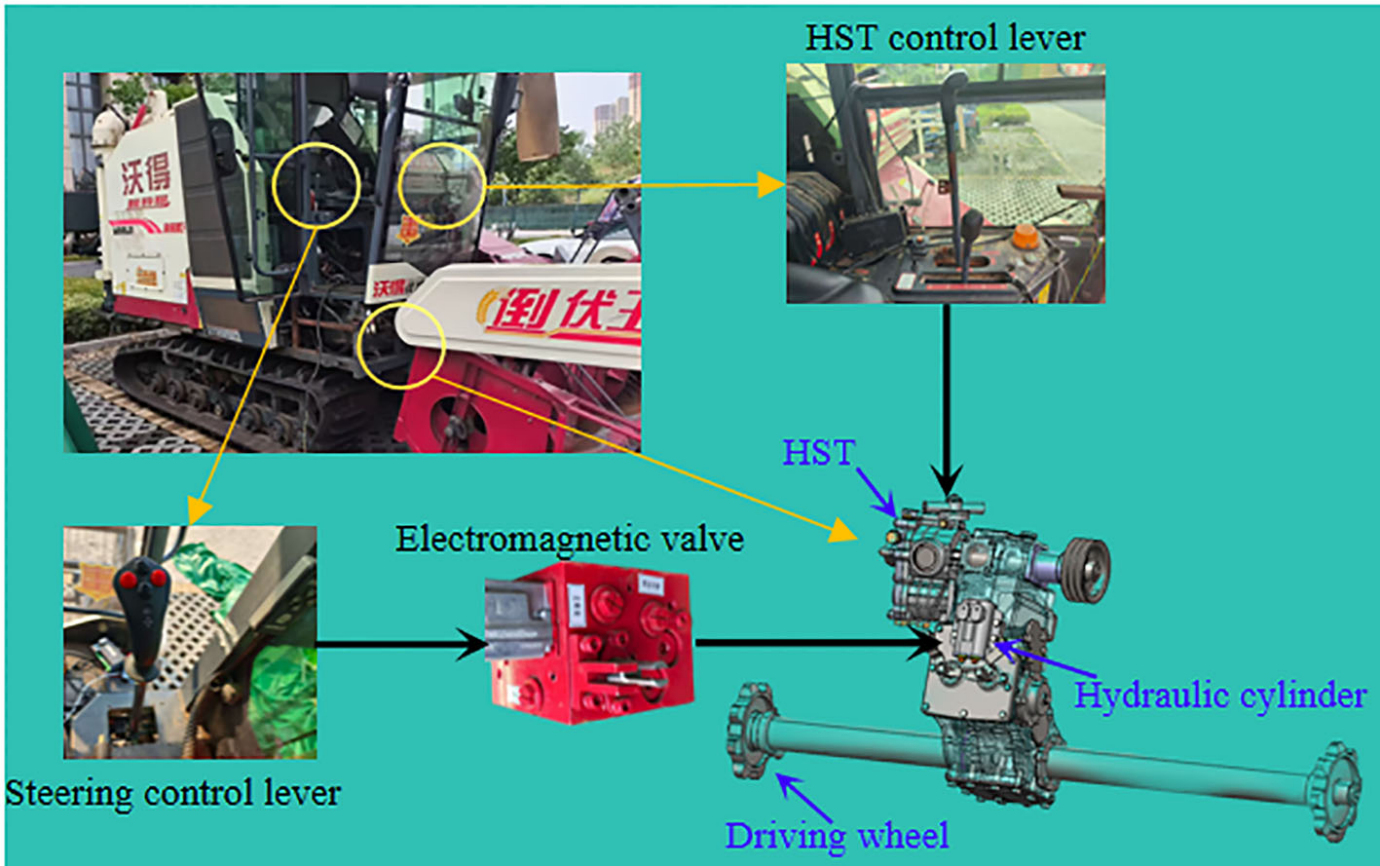
Schematic diagram of control system structure.

Tracked harvesters mainly achieve the forward, backward, and steering control of the single-side braking chassis system through HST. The system is mainly composed of a variable-displacement hydraulic pump, hydraulic motors, a control valve group, a hydraulic oil tank, and pipelines. This type of chassis regulates the steering cylinder through the steering valve, thereby achieving precise control of the single-side track clutch: when in - situ steering is required, the steering valve drives the steering cylinder to completely disengage the single - side clutch; while during differential steering, the steering valve controls the clutch to be in a semi-engaged state, and the turning action is completed through the speed difference between the two tracks. This not only ensures the power stability during straight-line driving but also improves the steering response speed under complex terrains. The turning radius of the harvester in the differential-steering state is not adjustable. This type of single-side braking and steering harvester has five steering modes: neutral, straight-driving, left and right *in-situ* steering, and left and right semi-engaged steering. The steering speed of the harvester increases with the increase of the vehicle speed, and when the steering speed is relatively fast, it will cause the harvester to jolt and jump as a whole. Therefore, during error adjustment, it is regulated in the form of semi-engaged steering. This provides a precise and reliable operation method. The HST steering schematic is shown in **Figure 3**.

#### 2.1.3 Autonomous navigation system software components

**Figure 4:**
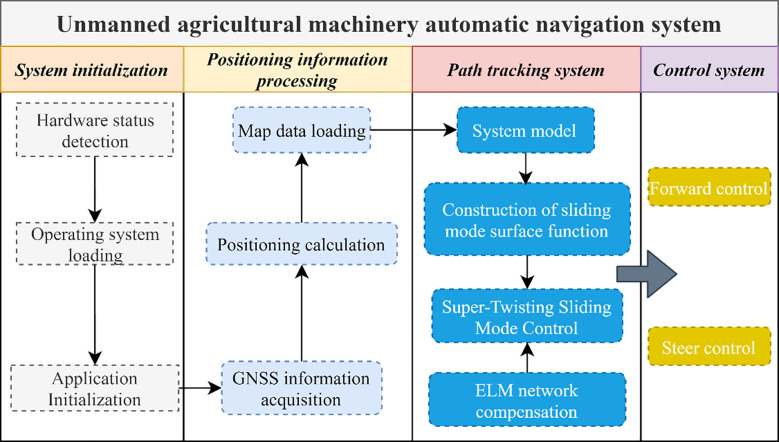
Structure of the automatic navigation software system.

The core of the perception and planning layer of the autonomous navigation software layered control system is a self-developed control platform operating on the host computer. Within this system, the key position coordinates of the harvester are defined, and a reference path is generated accordingly. Simultaneously, real-time data collected from the RTK-GNSS system is analyzed to determine the current position of the vehicle and to compute path-tracking errors, including lateral and heading deviations (Hu et al., 2025). In the decision-making and control layer, an STM32-based controller is used to deploy the proposed algorithm on the experimental platform. After receiving the path-tracking deviation data from the perception layer, the host computer executes the control algorithm. This layer is responsible for processing multi-sensor data from the harvester via serial communication and issuing control commands to the low-level controller. The control architecture is designed based on Lyapunov stability theory, and theoretical analysis confirms its global asymptotic convergence. Finally, the computed driving and steering commands are transmitted to the underlying control layer, whose main function is to implement closed-loop control of the steering angle and driving speed. This control structure forms the foundation for achieving path-tracking control of the vehicle. The overall system structure is shown in **Figure 4**.

### 2.2 Kinematic modelling of tracked harvesters

**Figure 5:**
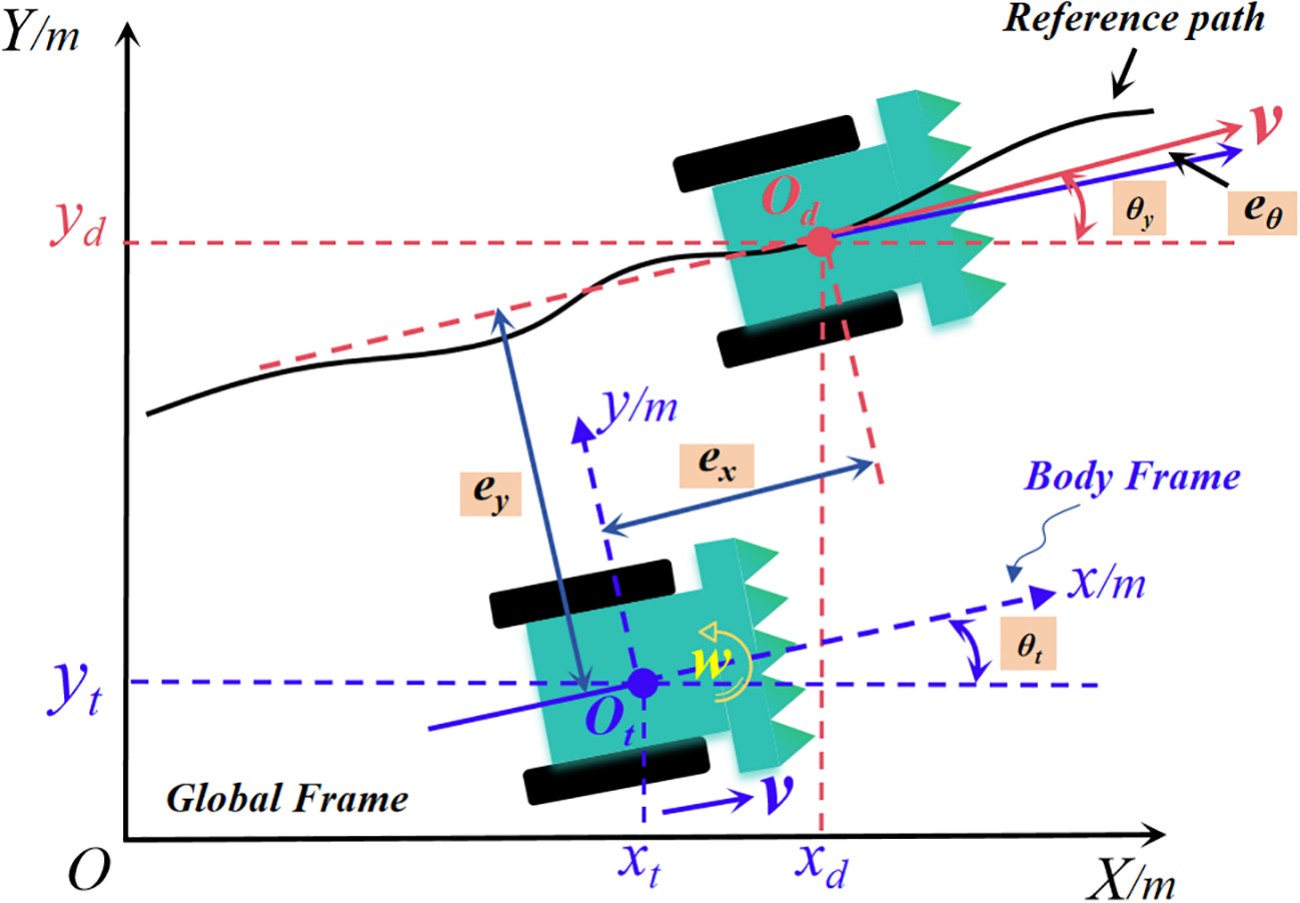
Schematic diagram of harvester path tracking.


Before designing the harvester path tracking control law, it is necessary to establish the vehicle kinematics model based on its actual working condition. In the modelling process, the vehicle is simplified as follows: the driving resistance of the tracked vehicle in the steering process is the same as during straight driving. The vehicle suspension and the track tensioning force during steering process are not considered. The origin of the dynamic coordinate system is coincident with the center of mass, and the simplified kinematic model of the tracked vehicle is shown in **Figure 5**. To better describe the error between the actual trajectory of the vehicle and the reference trajectory, a global coordinate system *XOY* is constructed, and the actual position of the vehicle at moment ‘t’ is denoted as *O_t_*. The corresponding position is written as $\left[x_{t},y_{t},\theta_{t}\right]$ the ideal position *O_d_*, and the corresponding position at this point is denoted as $\left[x_{d},y_{d},\theta_{d}\right]$ . Then the trajectory tracking dynamic error model can be obtained as:

(1) $$\{\begin{array}{c} x(\text{t}+1)=x(t)+v\cdot \cos(\theta (t))\cdot T_{s}\\ y(t+1)=y(t)+v\cdot \sin(\theta (t))\cdot T_{s}\\ \theta (t+1)=\theta (t)+\omega \cdot T_{s} \end{array}$$

(2) $$\left[\begin{array}{c} e_{x}\\ e_{y}\\ e_{\theta } \end{array}\right]=\left[\begin{array}{ccc} \cos\theta_{d} & \sin\theta_{d} & 0\\ -\sin\theta_{d} & \cos\theta_{d} & 0\\ 0 & 0 & 1 \end{array}\right]\left[\begin{array}{c} x_{t}-x_{d}\\ y_{t}-y_{d}\\ \theta_{t}-\theta_{d} \end{array}\right]$$

Where *V* represents the vehicle speed; *ω* represents the angular velocity; *Ts* represents the sampling time interval; *θ(t)* represents the vehicle heading angle at the current moment; *e_x_* and *e_y_* are the position coordinate errors, and *e_θ_* is the heading angle error. In the global coordinate system, the vehicle has a body coordinate system with its origin fixed at the center of mass of the vehicle. To achieve an effective path tracking effect, this study transforms the vehicle trajectory tracking problem into gradually iterating the lateral offset and heading deviation to 0 using the designed control law. So the lateral error and heading error as: ${\text{e}}_{y}=-(x_{t}-x_{d})\sin\theta_{d}+(y_{t}-y_{d})\cos\theta_{d},e_{\theta }=\theta_{t}-\theta_{d}$ .

## 3 Control scheme design and stability analysis

### 3.1 Path tracking sliding mode controller design

In automatic navigation systems, the purpose of path-tracking control is to eliminate lateral and heading deviations of the vehicle and ensure that it can accurately follow the pre-defined reference path. To achieve this, the present study adopts the generalized terminal sliding mode control framework to construct a robust sliding mode surface. This surface is specifically designed to ensure that both lateral and heading deviations converge to zero within a finite time during the tracking process. The design of sliding mode surface and controller is shown in Equations 2–5. The proposed generalized terminal sliding mode surface is formulated as follows:


(3) $$s=e_{\theta }+k_{1}|e_{y}{|}^{\alpha }sign(e_{y})+k_{2}|e_{\theta }{|}^{\beta }sign(e_{\theta })$$

where *k*_1_, *k*_2_ > 0, 0<*α*<1, *β* > 1, as can be seen by taking a derivative of it:


(4) $$\dot{s}={\dot{e}}_{\theta }+k_{1}\alpha |e_{y}{|}^{\alpha -1}{\dot{e}}_{y}+k_{2}\beta |e_{\theta }{|}^{\beta -1}{\dot{e}}_{\theta }$$

To address the challenges of path tracking disturbances in the harvester, particularly those arising from variations in reference path curvature, this study aims to mitigate the high-frequency oscillations typically observed in conventional sliding-mode control algorithms upon reaching the sliding-mode surface. Consequently, a STSMC (Sun et al., 2023) is implemented, and its design process is described below.


(5) $$\omega =\omega_{ref}-\lambda_{1}|s{|}^{1/2}sign(s)-\lambda_{2}\int_{0}^{t}sign(s)d\tau -\left(\gamma_{1}|s{|}^{\alpha }+\gamma_{2}|s{|}^{\beta }\right)sign(s)-\hat{d}$$

where $\hat{d}$ is the interference value estimated by the ELM, *W_ref_* is the desired angular velocity. *λ*_1_, *λ*_2_ > 0.

After bringing the control law to the first order derivatives of the sliding mode surface as (Equation 6):


(6) $$\begin{array}{l} \dot{s}=-\lambda_{1}|s{|}^{1/2}sign(s)-\lambda_{2}\int_{0}^{t}sign(s)d\tau -\left(\gamma_{1}|s{|}^{\alpha }+\gamma_{2}|s{|}^{\beta }\right)sign(s)-\hat{d}+k_{1}\alpha |e_{y}{|}^{\alpha -1}{\dot{e}}_{y}+\\ k_{2}\beta |e_{\theta }{|}^{\beta -1}\left[-\lambda_{1}|s{|}^{1/2}sign(s)-\lambda_{2}\int_{0}^{t}sign(s)d\tau -\left(\gamma_{1}|s{|}^{\alpha }+\gamma_{2}|s{|}^{\beta }\right)sign(s)-\hat{d}\right] \end{array}$$

The collation of Equation 6 gives:


(7) $$\begin{array}{l} \dot{s}=-\lambda_{1}\left(1+k_{2}\beta |e_{\theta }{|}^{\beta -1}\right)|s{|}^{1/2}sign(s)-\lambda_{2}\left(1+k_{2}\beta |e_{\theta }{|}^{\beta -1}\right)\int_{0}^{t}\sin(s)d\tau \\ -(1+k_{2}\beta |e_{\theta }{|}^{\beta -1})(\gamma_{1}|s{|}^{\alpha }+\gamma_{2}|s{|}^{\beta })sign(s)+k_{1}\alpha |e_{y}{|}^{\alpha -1}{\dot{e}}_{y}-\hat{d}(1+k_{2}\beta |e_{\theta }{|}^{\beta -1}) \end{array}$$

### 3.2 Extreme learning machine

In the design of STSMC, the upper bound of the aggregate perturbation in the path tracking deviation system needs to be known in advance. However, this upper bound is usually difficult to obtain. In engineering practice, to solve the problem, through the need to select a gain to suppress the unknown perturbation suppression, in this research study, the use of an extreme learning machine network is proposed to compensate for its perturbation, to reduce the jitter vibration phenomenon generated by the controller. The specific derivation is from Equations 8–15. Extreme Learning Machine (ELM) is an efficient single hidden layer feedforward neural network (SLFN) training method (Zou et al., 2022). For a single hidden-layer neural network, suppose there are N arbitrary samples $\left(X_{i},t_{i}\right)$ $t_{i}={\left[t_{i1},t_{i2},\cdots ,t_{im}\right]}^{T}\in R^{m}\;$ , the weights from the hidden layer to the output layer are $W={\left[W_{1},W_{2},\ldots ,W_{L}\right]}^{T}$ , the input weights are $\beta_{i}={\left[\beta_{i,1},\beta_{i,2},\cdots ,\beta_{i,n}\right]}^{T}$ , for a single hidden-layer neural network with L hidden-layer nodes can be represented as:


(8) $$\sum_{i=1}^{L}W_{i}g\left(\beta_{i}\cdot X_{j}+b_{i}\right)=o_{j},j=1,\cdots ,N$$

where g(x) is the activation function, bi is the bias of the hidden layer unit, and $W_{i}$ is the output weight;

The goal of single-hidden-layer neural network learning is to minimize the error in the output, which can be expressed as:


(9) $$\sum_{j=1}^{N}||o_{j}-t_{j}||=0$$

It can be expressed in terms of a matrix as:


(10) HW=T

where H is the output of the hidden layer node, W is the output weight, and T is the desired output.


(11) $$H(\beta_{1},\cdots ,\beta_{L},b_{1},\cdots ,b_{L},X_{1},\cdots ,X_{L})==={\left[\begin{array}{ccc} g(\beta_{1}.X_{1}+b_{1}) & \cdots & g(\beta_{L}.X_{1}+b_{L})\\ \vdots & \cdots & \vdots \\ g(\beta_{1}.X_{N}+b_{1}) & \cdots & g(\beta_{L}.X_{N}+b_{L}) \end{array}\right]}_{N\times L}$$

where $W={\left[W_{1}^{T}\ldots W_{L}^{T}\right]}^{L\times m}T={\left[T_{1}^{T}\ldots T_{N}^{T}\right]}^{N\times m},$ for training a single hidden layer neural network and thus obtaining estimates of each parameter, makes:


(12) $$∥H\left({\hat{\beta }}_{i},{\hat{b}}_{i}\right){\hat{W}}_{i}-T||=\underset{\beta ,b,W}{\min}||H\left(\beta_{i},b_{i}\right)|W_{i}-T||$$

RLS-ELM uses online-recursive least squares to update the output weights with faster convergence and adaptability to time-varying systems. The output weights of conventional ELM are updated by minimizing offline or online gradient descent. The output weights of RLS-ELM are updated as shown below:


(13) $$W(k)=W(k-1)+K(k)\left[d(k)-H^{T}(k)W(k-1)\right]$$

Its gain matrix is:


(14) $$K(k)=\frac{P(k-1)H(k)}{\eta +H^{T}(k)P(k-1)H(k)}$$

The formula for updating the covariance matrix is shown below:


(15) $$P(k)=\frac{1}{\eta }\left[P(k-1)-K(k)H^{T}(k)P(k-1)\right]$$

where λ ∈ (0,1] is the forgetting factor, which regulates the weights of the historical data.

**Figure 6:**
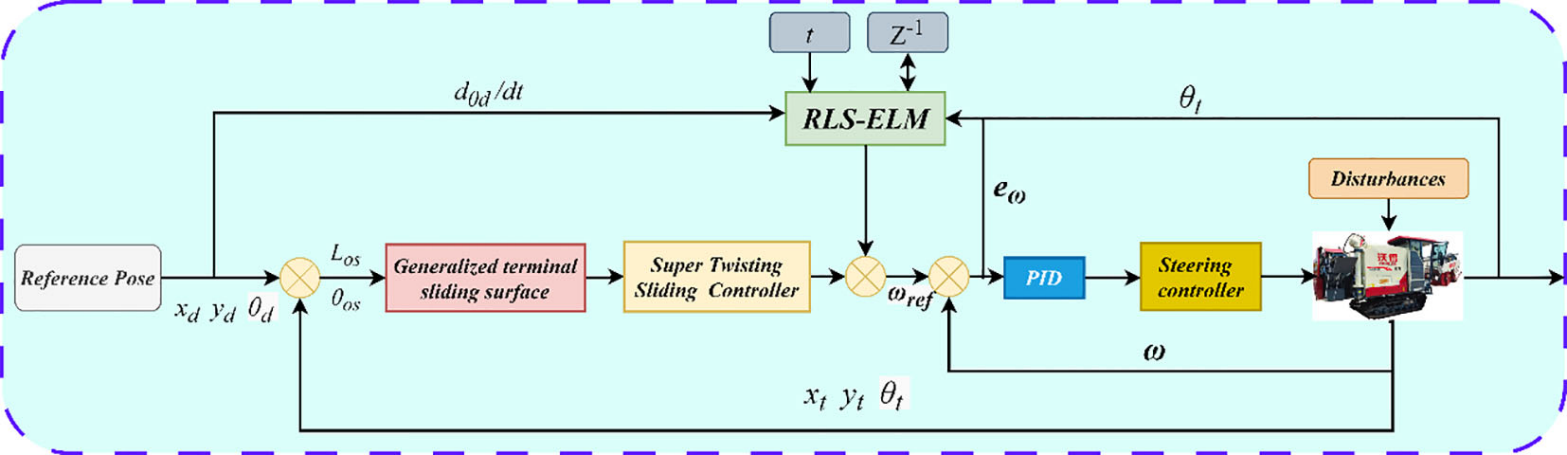
Schematic diagram of control logic for the system.

Traditional ELM relies on batch data for offline training and needs to compute the generalized inverse matrix of the Hermitian matrix at one time. In this paper, we design the RLS-ELM using the same framework as the traditional network, which combines the efficient online updating capability of the recursive least squares method with the fast initialization feature of ELM, making it suitable for real-time modelling and control in dynamic environments. At its core, it avoids repeated computation of the generalized inverse matrix by recursive updating of the covariance matrix, which significantly reduces the computational complexity and leads to better updating of the output weights. The control schematic of the system is shown in **Figure 6**.

### 3.3 Controller stability analysis

In this research, the total number of unknown disturbances and their time derivatives are bounded (Ji et al., 2023a), $|d|\leq A_{0}$ $|\dot{d}|\leq A_{1}$ , where A_0_ and A_1_ are two effective constants. The derivation of stability control is shown in Equations 16–34. It is worth noting that the unknown perturbation is caused by the presence of poor road smoothness, sensor noise and the vehicle’s load variations in the unstructured environment in which the tracked vehicle is travelling, which always affects the path tracking system and the perturbation is usually finite, so that it can be regarded as a bounded term, and similar perturbation assumptions in real scenarios are also found in the related literature. The actual travelling speed of the tracked harvester is usually low, which makes the unknown disturbance change slowly. Therefore, the derivative of the unknown disturbance can also be considered bounded. Suppose the nonlinear system is as follows (Ji et al., 2022):


(16) $$\dot{x}=f(x),x(0)=x_{0}$$

where the state variable of the system *x* ∈ *Rn* and *f*(*x*) denotes a nonlinear continuous function. If this nonlinear system can be stabilized in finite time and the system stabilization time *t*1 is independent of the system initial condition *x*0,


(17) $$\underset{t\to t_{1}}{\lim}||x(t)||=0$$

and *x*(*t*) = 0 when time *t* > *t*_1_, which satisfies the fixed-time stability condition. Assume that a Lyapunov candidate exists and is satisfied (Ding et al., 2022):


(18) $$\dot{V}\leq -\psi_{1}V^{\zeta_{1}}-\psi_{2}V^{\zeta_{2}}+\Delta$$

Where $\psi_{1}$ and $\psi_{2}$ ∈ *R*, 0< $\zeta_{1}$ <1< $\zeta_{2}$ , 0 < Δ < ∞, 0 < θ <1, then the state of this nonlinear system is bounded by the following equation (Li et al., 2023):


(19) $$\left\{\underset{t\to T}{\lim}x|V\leq \min\left\{{\left(\frac{\Delta }{(1-\theta )\psi_{1}}\right)}^{1/\zeta_{1}},{\left(\frac{\Delta }{(1-\theta )\psi_{2}}\right)}^{1/\zeta_{2}}\right\}\right\}$$

Considering the scalar differential equation (Ji et al., 2023b):


(20) $$\dot{z}=-\lambda |z{|}^{\alpha }sign\left(z\right)-\mu |z{|}^{\gamma }sign\left(z\right)$$

which equation has the same fixed-time convergence upper bound *T*_0_ for any initial condition *z*(0):


(21) $$T\leq T_{0}(\alpha ,\gamma ,\lambda ,\mu )=\frac{1}{\lambda (\alpha -1)}+\frac{1}{\mu (1-\gamma )}$$

Stability is a prerequisite for the normal operation of control systems. In practical engineering, systems are often nonlinear and time-varying. The Lyapunov method directly analyzes the stability of nonlinear systems by constructing Lyapunov functions, without the need to solve the system’s differential equations. It directly determines whether the system has converged to an equilibrium state through the sign properties of functions and their derivatives, providing theoretical support for the control design of complex systems (Moreno and Osorio, 2012). To ensure the stability and effectiveness of the controller design, the stability of the controller designed in this research is proved by constructing the Lyapunov function. The constructed Lyapunov function is shown below:


(22) $$V=\frac{1}{2}s^{2}+\frac{1}{2}{\tilde{W}}^{T}P^{-1}\tilde{W}$$

The derivation of this can be shown:


(23) $$\dot{V}=s\dot{s}+{\tilde{W}}^{T}P^{-1}\dot{\tilde{W}}+\frac{1}{2}{\tilde{W}}^{T}{\dot{P}}^{-1}\tilde{W}$$

(24) $$\begin{array}{l} \dot{V}=-\lambda_{1}\left(1+k_{2}\beta |e_{\theta }{|}^{\beta -1}\right)|s{|}^{3/2}-\lambda_{2}\left(1+k_{2}\beta |e_{\theta }{|}^{\beta -1}\right)s\int_{0}^{t}sign(s)\;d\tau -\\ \left(1+k_{2}\beta |e_{\theta }{|}^{\beta -1}\right)\left(\gamma_{1}|s{|}^{\alpha }+\gamma_{2}|s{|}^{\beta }\right)-\hat{d}\left(1+k_{2}\beta |e_{\theta }{|}^{\beta -1}\right)s+k_{1}\alpha |e_{y}{|}^{\alpha -1}s{\dot{e}}_{y}\\ +{\tilde{W}}^{T}P^{-1}\dot{\tilde{W}}+\frac{1}{2}{\tilde{W}}^{T}{\dot{P}}^{-1}\tilde{W} \end{array}$$

Where the actual perturbation in the perturbation estimation error *d* consists of the estimation of the ELM $\hat{d}$ as well as the approximation error ϵ , as follows:


(25) $$d=\hat{d}+\epsilon =W^{T}H+\epsilon$$

Where the approximation error of the ELM is bounded $|\epsilon |\leq \epsilon_{0}$ , $\epsilon_{0}$ is a constant greater than 0. Due to $\dot{\tilde{W}}=-\dot{W}$ substituting this into the RLS update law:


(26) $$\{\begin{array}{c} \dot{W}=K(d-H^{T}W)=K(H^{T}\tilde{W}+\epsilon )\\ \dot{\tilde{W}}=-K\left(H^{T}\tilde{W}+\epsilon \right) \end{array}$$

where $W^{*}$ is the desired weight, $\tilde{W}=W^{*}-W$ .


(27) $$\begin{array}{l} \dot{V}=-\lambda_{1}\left(1+k_{2}\beta |e_{\theta }{|}^{\beta -1}\right)|s{|}^{3/2}-\lambda_{2}\left(1+k_{2}\beta |e_{\theta }{|}^{\beta -1}\right)s\int_{0}^{t}sign(s)\;d\tau -\\ \left(1+k_{2}\beta |e_{\theta }{|}^{\beta -1}\right)\left(\gamma_{1}|s{|}^{\alpha }+\gamma_{2}|s{|}^{\beta }\right)-H{\tilde{W}}^{T}\left(1+k_{2}\beta |e_{\theta }{|}^{\beta -1}\right)s+\\ k_{1}\alpha |e_{y}{|}^{\alpha -1}s{\dot{e}}_{y}+\frac{1}{2}{\tilde{W}}^{T}(\eta P^{-1}+HH^{T})\tilde{W}-{\tilde{W}}^{T}P^{-1}KH^{T}\tilde{W}-{\tilde{W}}^{T}P^{-1}K\epsilon \end{array}$$

where ${\dot{P}}^{-1}=\eta P^{-1}+HH^{T}$ it can be seen from the above Equation 1 $+k_{2}\beta |e_{\theta }{|}^{\beta -1}\geq 1$ K=PH(x)/η . Therefore, Equation 27. can be scaled to:


(28) $$\begin{array}{l} \dot{V}\leq -\left(1+k_{2}\beta |e_{\theta }{|}^{\beta -1}\right)\left(\gamma_{1}|s{|}^{\alpha +1}+\gamma_{2}|s{|}^{\beta +1}\right)-H{\tilde{W}}^{T}\left(1+k_{2}\beta |e_{\theta }{|}^{\beta -1}\right)+\frac{1}{2}{\tilde{W}}^{T}(\eta P^{-1}+HH^{T})\tilde{W}\\ -{\tilde{W}}^{T}P^{-1}KH^{T}\tilde{W}-{\tilde{W}}^{T}P^{-1}K\epsilon \\ =-\left(1+k_{2}\beta |e_{\theta }{|}^{\beta -1}\right)\left(\gamma_{1}|s{|}^{\alpha +1}+\gamma_{2}|s{|}^{\beta +1}\right)-H{\tilde{W}}^{T}\left(1+k_{2}\beta |e_{\theta }{|}^{\beta -1}\right)s+\frac{1}{2}{\tilde{W}}^{T}|\eta P^{-1}+HH^{T})\tilde{W}\\ -\frac{1}{\eta }{\tilde{W}}^{T}P^{-1}PHH^{T}\tilde{W}-\frac{1}{\eta }{\tilde{W}}^{T}P^{-1}PH\epsilon \\ =-\left(1+k_{2}\beta |e_{\theta }{|}^{\beta -1}\right)\left(\gamma_{1}|s{|}^{\alpha +1}+\gamma_{2}|s{|}^{\beta +1}\right)-H{\tilde{W}}^{T}\left(1+k_{2}\beta |e_{\theta }{|}^{\beta -1}\right)s+\frac{1}{2}{\tilde{W}}^{T}|\eta P^{-1}+HH^{T})\tilde{W}\\ -\frac{1}{\eta }{\tilde{W}}^{T}HH^{T}\tilde{W}-\frac{1}{\eta }{\tilde{W}}^{T}H\epsilon \\ =-\left(1+k_{2}\beta |e_{\theta }{|}^{\beta -1}\right)\left(\gamma_{1}|s{|}^{\alpha +1}+\gamma_{2}|s{|}^{\beta +1}\right)-H{\tilde{W}}^{T}\left(1+k_{2}\beta |e_{\theta }{|}^{\beta -1}\right)s\\ +{\tilde{W}}^{T}\left(\frac{1}{2}\eta P^{-1}+(\frac{1}{2}-\frac{1}{\eta })HH^{T}\right)\tilde{W}-\frac{1}{\eta }{\tilde{W}}^{T}H\epsilon \end{array}$$

By using Young’s inequality, Equation 28 can be further scaled into:


(29) $$\begin{array}{l} \dot{V}\leq -\left(1+k_{2}\beta |e_{\theta }{|}^{\beta -1}\right)\left(\gamma_{1}|s{|}^{\alpha +1}+\gamma_{2}|s{|}^{\beta +1}\right)-H{\tilde{W}}^{T}\left(1+k_{2}\beta |e_{\theta }{|}^{\beta -1}\right)s\\ +{\tilde{W}}^{T}\left(\frac{1}{2}\eta P^{-1}+(\frac{1}{2}-\frac{1}{\eta })HH^{T}\right)\tilde{W}-\frac{1}{\eta }{\tilde{W}}^{T}H\epsilon \\ \leq -\left(1+k_{2}\beta |e_{\theta }{|}^{\beta -1}\right)\left(\gamma_{1}|s{|}^{\alpha +1}+\gamma_{2}|s{|}^{\beta +1}\right)+\frac{1}{2}\left(1+k_{2}\beta |e_{\theta }{|}^{\beta -1}\right){\text{s}}^{2}+\left(1+k_{2}\beta |e_{\theta }{|}^{\beta -1}\right)||H|{|}^{2}||\tilde{W}|{|}^{2}\\ +{\tilde{W}}^{T}\left(\frac{1}{2}\eta P^{-1}+(\frac{1}{2}-\frac{1}{\eta })HH^{T}\right)\tilde{W}+\frac{1}{2\eta }||H|{|}^{2}||\tilde{W}|{|}^{2}+\frac{1}{2\eta }\epsilon_{\;0}^{2}\\ \leq -\left(1+k_{2}\beta |e_{\theta }{|}^{\beta -1}\right)\left(\gamma_{1}|s{|}^{\alpha +1}+\gamma_{2}|s{|}^{\beta +1}\right)+\left(1+\frac{1}{\eta }+k_{2}\beta |e_{\theta }{|}^{\beta -1}\right)||H|{|}^{2}||\tilde{W}|{|}^{2}\\ +{\tilde{W}}^{T}\left(\frac{1}{2}\eta P^{-1}+(\frac{1}{2}-\frac{1}{\eta })HH^{T}\right)\tilde{W}+\frac{1}{2\eta }\epsilon_{\;0}^{2}\\ \leq -\left(1+k_{2}\beta |e_{\theta }{|}^{\beta -1}\right)\left(\gamma_{1}|s{|}^{\alpha +1}+\gamma_{2}|s{|}^{\beta +1}\right)+\left(1+k_{2}\beta |e_{\theta }{|}^{\beta -1}\right)||H|{|}^{2}||\tilde{W}|{|}^{2}\\ +\frac{1}{2}\eta P^{-1}{\tilde{W}}^{T}\tilde{W}+\frac{1}{2}HH^{T}{\tilde{W}}^{T}\tilde{W}+\frac{1}{2\eta }\epsilon_{\;0}^{2}\\ \leq -\left(1+k_{2}\beta |e_{\theta }{|}^{\beta -1}\right)\left(\gamma_{1}|s{|}^{\alpha +1}+\gamma_{2}|s{|}^{\beta +1}\right)+\left(\frac{3}{2}+k_{2}\beta |e_{\theta }{|}^{\beta -1}\right)||H|{|}^{2}||\tilde{W}|{|}^{2}\\ +\frac{1}{2}\eta P^{-1}||\tilde{W}|{|}^{2}+\frac{1}{2\eta }\epsilon_{\;0}^{2}\\ \leq -\left(1+k_{2}\beta |e_{\theta }{|}^{\beta -1}\right)\left(\gamma_{1}|s{|}^{\alpha +1}+\gamma_{2}|s{|}^{\beta +1}\right)+\frac{1}{2}\eta P^{-1}||\tilde{W}|{|}^{2}+\frac{1}{2\eta }\epsilon_{\;0}^{2} \end{array}$$

By as Jensen’s inequality, Equation 29 can be further scaled into:


(30) $$\begin{array}{l} \dot{V}\leq -\left(1+k_{2}\beta |e_{\theta }{|}^{\beta -1}\right)\left(\gamma_{1}|s{|}^{\alpha +1}+\gamma_{2}|s{|}^{\beta +1}\right)+\frac{1}{2}\eta P^{-1}||\tilde{W}|{|}^{2}+\frac{1}{2\eta }\epsilon_{\;0}^{2}\\ \leq -\left(1+k_{2}\beta |e_{\theta }{|}^{\beta -1}\right)\left(\gamma_{1}|s{|}^{\alpha +1}+\gamma_{2}|s{|}^{\beta +1}+\gamma_{1}{\left({\tilde{W}}^{T}P^{-1}\tilde{W}\right)}^{\frac{\alpha +1}{2}}+\gamma_{2}2^{\beta }{\left({\tilde{W}}^{T}P^{-1}\tilde{W}\right)}^{\frac{\beta +1}{2}}\right)\\ +\gamma_{1}\left(1+k_{2}\beta |e_{\theta }{|}^{\beta -1}\right){\left({\tilde{W}}^{T}P^{-1}\tilde{W}\right)}^{\frac{\alpha +1}{2}}+\gamma_{2}2^{\beta }\left(1+k_{2}\beta |e_{\theta }{|}^{\beta -1}\right){\left({\tilde{W}}^{T}P^{-1}\tilde{W}\right)}^{\frac{\beta +1}{2}}+\frac{1}{2}\eta P^{-1}||\tilde{W}|{|}^{2}+\frac{1}{2\eta }\epsilon_{\;0}^{2}\\ \leq -\left(1+k_{2}\beta |e_{\theta }{|}^{\beta -1}\right)\left(2\gamma_{1}V^{\frac{\alpha +1}{2}}+\gamma_{2}V^{\frac{\beta +1}{2}}\right)+\gamma_{1}\left(1+k_{2}\beta |e_{\theta }{|}^{\beta -1}\right)+\gamma_{1}\left(1+k_{2}\beta |e_{\theta }{|}^{\beta -1}\right){\left(P^{-1}||\tilde{W}|{|}^{2}\right)}^{\frac{\alpha +1}{2}}\\ +\gamma_{2}2^{\beta }\left(1+k_{2}\beta |e_{\theta }{|}^{\beta -1}\right){\left(P^{-1}||\tilde{W}|{|}^{2}\right)}^{\frac{\beta +1}{2}}+\frac{1}{2}\eta P^{-1}||\tilde{W}|{|}^{2}+\frac{1}{2\eta }\epsilon_{\;0}^{2} \end{array}$$

Let L_min_ (P) be the smallest eigenvalue of matrix P, then it can be further calculated along Equation 30:


(31) $$\begin{array}{l} \dot{V}\leq -2\gamma_{1}\left(1+k_{2}\beta |e_{\theta }{|}^{\beta -1}\right)V^{\frac{\alpha +1}{2}}-\gamma_{2}\left(1+k_{2}\beta |e_{\theta }{|}^{\beta -1}\right)V^{\frac{\beta +1}{2}}+2\gamma_{1}{\left(\frac{||\tilde{W}|{|}^{2}}{L_{\min}(P)}\right)}^{\frac{\alpha +1}{2}}\\ +\gamma_{2}{\left(\frac{||\tilde{W}|{|}^{2}}{L_{\min}(P)}\right)}^{\frac{\beta +1}{2}}+\frac{1}{2}\eta \frac{||\tilde{W}|{|}^{2}}{L_{\min}(P)}+\frac{1}{2\eta }\epsilon_{\;0}^{2} \end{array}$$

Equation 27 can be obtained:


(32) $$\dot{V}\leq -\psi_{1}V^{\frac{\alpha +1}{2}}-\psi_{2}V^{\frac{\beta +1}{2}}+\Delta$$

Among them: $\psi_{1}=2\gamma_{1}\left(1+k_{2}\beta |e_{\theta }{|}^{\beta -1}\right)$ , $\psi_{2}=\gamma_{2}\left(1+k_{2}\beta |e_{\theta }{|}^{\beta -1}\right)$ , $\Delta =2\gamma_{1}{\left(\frac{||\tilde{W}|{|}^{2}}{L_{\min}(P)}\right)}^{\frac{\alpha +1}{2}}+\gamma_{2}{\left(\frac{||\tilde{W}|{|}^{2}}{L_{\min}(P)}\right)}^{\frac{\beta +1}{2}}+\frac{1}{2}\eta \frac{||\tilde{W}|{|}^{2}}{L_{\min}(P)}+\frac{1}{2\eta }\epsilon_{\;\;0}^{2}$ Thus, the sliding mode variable s will converge to an arbitrarily small neighborhood of the origin in a fixed time limited by a constant:


(33) $$T\leq \frac{1}{\psi_{1}(\frac{\alpha +1}{2}-1)}+\frac{1}{\psi_{2}(1-\frac{\beta +1}{2})}$$

The convergence time is independent of the initial path tracking offset, thus completing the stability proof of the controller proposed in this study (Zuo, 2015). The convergence region r is:


(34) $$r=\left\{\underset{t\to T}{\lim}x|V\leq \min\left\{{\left(\frac{\Delta }{(1-\theta )\psi_{1}}\right)}^{\frac{2}{\alpha +1}},{\left(\frac{\Delta }{(1-\theta )\psi_{2}}\right)}^{\frac{2}{\beta +1}}\right\}\right\}$$

Compared with the existing methods, the control scheme designed has the following advantages: (1) a fixed-time generalized terminal sliding mode surface is designed, which significantly improves the convergence speed, robustness and anti-interference ability of the system through the nonlinear structural design and the convergence time decoupling mechanism; (2) the super-twisting control law significantly improves the control accuracy and dynamic response performance under the complex perturbation by utilizing the higher-order sliding mode technique;(3) The RLS-ELM estimator is used to update the uncertainty boundary, and the RLS algorithm updates the weight matrix by recursive formulae, avoiding the repeated inverse operation of the matrix under the traditional ELM, reducing the computational volume significantly, and achieving real-time and accurate estimation of the dynamic and complex uncertainty boundary.

In the ELM-AFGST control strategy proposed in this article, the parameter selection criteria for the proposed control are given as follows: (i) *k*_1_ and *k*_2_ in formula (3) are the lateral error gain and heading error gain, respectively. The larger the values of these parameters, the faster the convergence speed, but the larger the control signal caused. During the experiment, appropriate parameters are manually adjusted to obtain them; *α* and *β* are related to the upper bound of fixed time convergence. Although larger values shorten the stable time value, they can cause chattering in the controller and affect its performance; (ii) In formula (5), *λ*_1_ and *λ*_2_ are the sliding mode approach gain and integral compensation gain, respectively, which determine the approach speed of the sliding mode surface and eliminate steady-state errors. However, larger values can cause significant chattering during the convergence process, thereby affecting control performance. Therefore, it is necessary to choose appropriate coefficients. *α* and *β* are related to the upper bound of fixed-time convergence. Although larger values shorten the stable time value, they can cause chattering in the controller and affect its performance; (iii) RLS-ELM updates the uncertainty bound and relaxes the requirement for upper bound information in traditional sliding mode control. As the number of hidden layer nodes increases, ELM can approximate the true value with arbitrary precision. However, when the number of hidden layer nodes is too large, it can lead to an increase in the probability of feature diversity due to random weights and a decrease in computation speed. Therefore, in edge devices or real-time systems, it is necessary to balance model performance and computing resources and choose a reasonable L value.

## 4 Analysis of simulation and field experiment results

### 4.1 Analysis of simulation experiment results

To verify the effectiveness of the ELM-AFGST control strategy proposed, relevant simulation experiments are necessary. The control system simulation is carried out in the MATLAB environment, and to make the comparative simulation more comprehensive and convincing, two different interference operation environments are considered. In the simulation comparison experiment, the simulated paths are the common “S” shaped curves and multi-curved paths in the driving process of agricultural machines (Zhang et al., 2025). The “S” shaped curve, which consists of straight and curved road features, was selected as the reference operating path for control strategy validation in both cases. It is essential to ensure vehicle stability while driving on a predetermined trajectory. In the simulation, the initial coordinate position and heading angle of the vehicle are set to [2, –1, 0], and the starting point of the reference operation path is set to the coordinate origin. In addition, to test the robust performance of the control scheme designed, disturbances are introduced and set to: $\rho =6V_{t}\cos(\theta_{os})\sin(t)$ . The different controller parameters are shown in **Table 2**.

**Table 2:** Parameters of different controllers.

Control scheme	Parameters
PID	*k_p_* = 5, *k_i_* = 0.1, *k_d_* = 15
Pure Pursuit	*L*= 6, *k_y_* = 15, *k_θ_* = 30
FTSMC	*k*_1_ = 1.2, *α* = 0.7, *λ*_1_ = 10, *λ*_2_ = 10
AFGST	*k*_1_ = 150, *k*_2_ = 40, *λ*_1_ = 5, *λ*_2_ = 4, *α* = 0.8, *β*= 1.7
ELM-AFGST	*k*_1_ = 150, *k*_2_ = 40, *λ*_1_ = 5, *λ*_2_ = 4, *α* = 0.8, *β*= 1.7, *L*=20

#### 4.1.1 Comparison of the effects of different speeds

**Figure 7:**
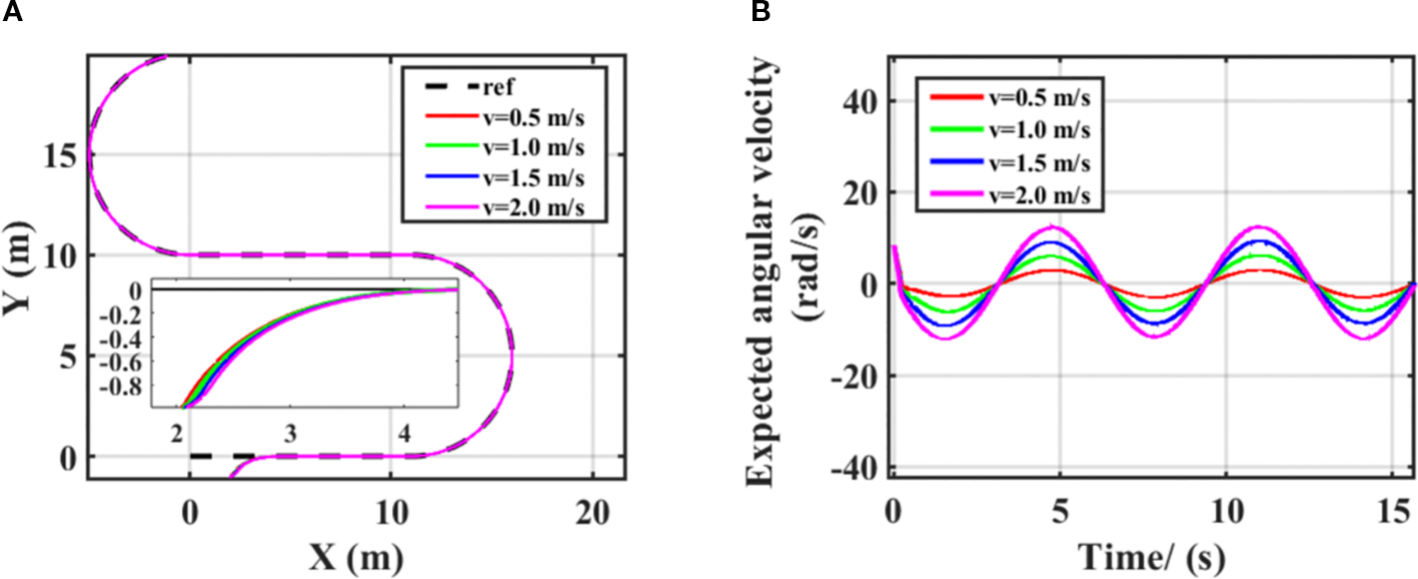
Control effects at different vehicle speeds **(A)** different trajectories, **(B)** different expected angular velocities.

The core goal of SMC is to quickly and stably converge the system state to the sliding surface, that is, to force the system to converge to a state with zero error through the sliding surface, thus achieving tracking of the reference trajectory and robustness to disturbances and parameter changes. During the movement of the harvester, as the speed changes, the dynamic parameters of the system, such as inertia and damping, will also change accordingly (increasing at high speeds and decreasing at low speeds). If the expected angular velocity is not adjusted with speed, the sliding surface may deviate from the optimal trajectory due to system parameter drift, resulting in increased tracking error or control oscillation. The steering system of the harvester has speed-related damping characteristics: as the speed increases, the damping of the steering mechanism will increase. Specifically, when the harvester is driving at high speed, the system sends the same control command, and the actual angular velocity output will decrease. Increasing the expected angular velocity at this time is essentially by increasing the command strength, offsetting the output attenuation caused by damping growth, and ensuring that the actual angular velocity can reach the required value for tracking the path; When the vehicle is driving at low speeds, the damping is small, the steering flexibility is high, and a small expected angular velocity can meet the response requirements. If the command is too large, it may even lead to overshoot. This adjustment enables the sliding mode controller to maintain stable tracking accuracy even when the speed dynamically changes. The angular velocity output results of the controller at different speeds are shown in **Figure 7**. As the vehicle speed increases, the expected angular velocity of the vehicle will also increase. When the vehicle speed decreases, the expected angular velocity of the vehicle will also decrease. This reflects the robustness of the sliding mode controller designed for different vehicle speeds, enabling the harvester to stably travel along the preset path during path tracking. Therefore, in the subsequent simulation, in order to balance efficiency and control effect, the simulation and experimental speed were set to 1m/s.

#### 4.1.2 Comparison of the effect of different controller algorithms

**Figure 8:**
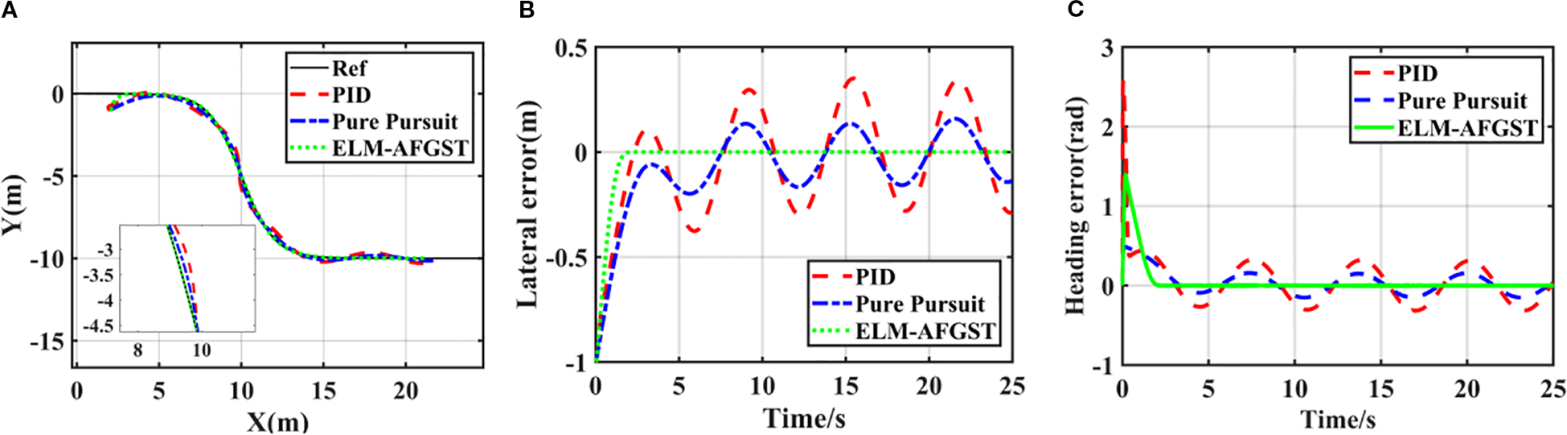
Multi-curved path tracking results **(A)** different trajectories, **(B)** lateral error, **(C)** heading error.

Under the “S” curve simulation, the linear controller, represented by the PID algorithm, the model control algorithm, represented by the pure tracking algorithm, and the sliding mode controller designed are used for comparison in the experiment. The simulation results for path tracking are shown in **Figure 8**, and the path comparison results are presented in **Figure 8A**. From the figure, it can be observed that the use of the ELM-AFGST controller significantly reduces overshooting during path tracking and improves the vehicle’s tracking accuracy. Considering that vehicles often encounter turning and other maneuvering scenarios during actual driving, the ELM-AFGST controller proposed can effectively improve the driving efficiency of agricultural machines. The comparative results of the lateral deviation and heading deviation simulations are shown in **Figures 8B, C**, from which it can be seen that the tracking error is the most stable during the path tracking process using the ELM-AFGST controller, which exhibits good robustness and the ability to suppress oscillations, which means that the vehicle can follow the desired path stably. It is worth noting that the PID controller has a slower response because it does not have a good anti-interference ability itself. The pure tracking algorithm is complex to obtain the optimal forward-looking distance because its tracking performance relies heavily on the selection of the forward-looking distance, so it produces large fluctuations in the path tracking when they are finally obtained, and the oscillations are obvious in the middle and late stages, and the stability is poor. Additionally, the simulation results show that in practical agricultural applications, the steady-state tracking error can be reduced by adjusting the control gain appropriately. However, a too large control gain may induce oscillations in the control signal.

**Figure 9:**
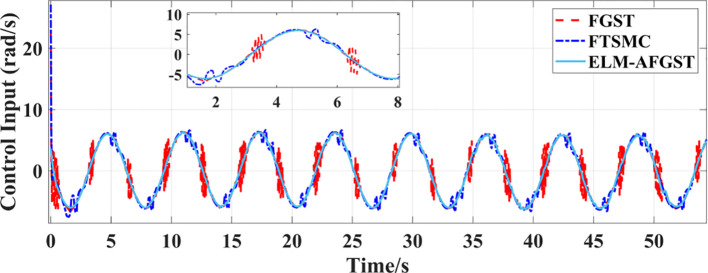
Input diagram of angular velocity control for path tracking system.

To further investigate the effectiveness of the ELM-AFGST controller proposed in this study to control the agricultural machine to track the multi-curved reference path consisting of straight lines and circular arcs. The angular velocity control input diagram of the path tracking system is shown in **Figure 9**. From **Figure 9**, it can be seen that in the path tracking control scenario, the FGST control input exhibits a certain degree of regular fluctuation. However, from the local magnified image, it can be seen that there are still small high-frequency vibrations in some periods; The fluctuation frequency and amplitude of the FTSMC control input are prominent, with obvious oscillation characteristics. This is due to the hard switching characteristic of the sign function in traditional terminal sliding mode control, which can easily cause frequent jumps in the control signal and impose a significant burden on the actuator, increasing the risk of mechanical vibration; Although there is some fluctuation in the ELM-AFGST control input, it is less compared to other controllers, reflecting the effect of RLS-ELM real-time disturbance compensation and integral smoothing of super distortion control law, which to some extent suppresses high-frequency chattering.

**Figure 10:**
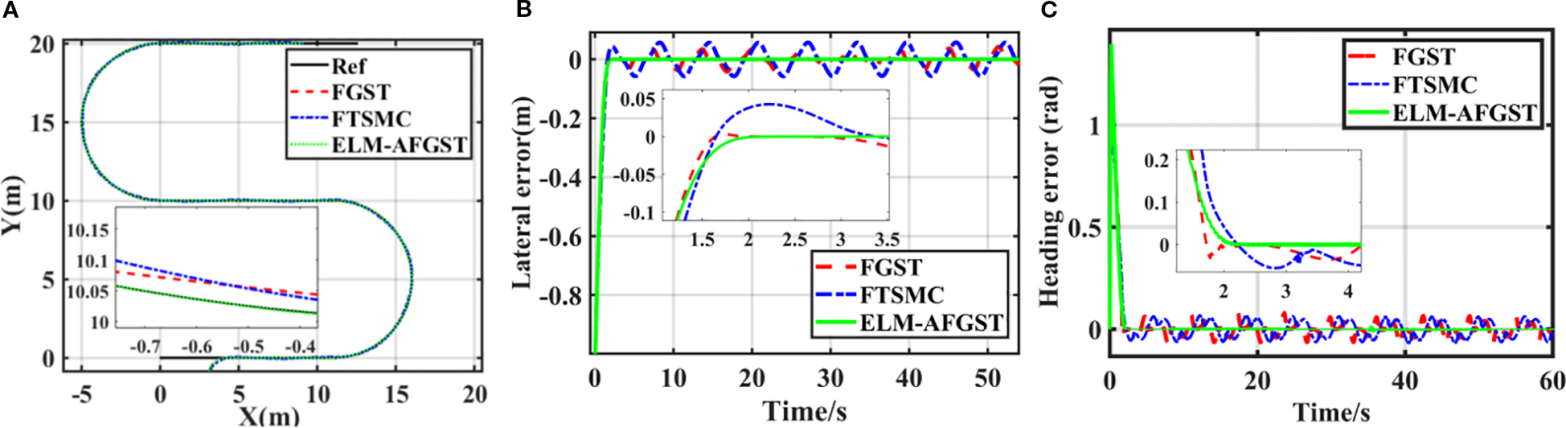
S-shaped path tracking results **(A)** different trajectories, **(B)** lateral error, **(C)** heading error.

The path tracking simulation results are shown in **Figure 10**. **Figure 10A** shows the actual results of the three controllers for tracking the path, and **Figures 10B, C** show the lateral error and heading error of the controls of the three controllers. From **Figures 10B, C**, it can be seen that the lateral deviation and heading error converge to zero quickly with the proposed ELM-AFGST controller, which is crucial for enhancing the tracking capabilities of autonomous agricultural vehicles. The system control stabilization time of the proposed ELM-AFGST controller and FGST controller is about 2 s, and the system control convergence time of the FTSMC controller is about 3.2 s. From the arrival system stabilization times of the three controllers, FTSMC, FGST, and ELM-AFGST, it can be seen that the proposed ELM-AFGST controller exhibits faster transient stability than the other two. The transient response speed of the proposed ELM-AFGST controller is faster than the other two controllers, and the steady-state average heading error is 0.002 rad/s. The error curves after path tracking under the control of the FTSMC controller and the FGST controller exhibit a more pronounced chattering phenomenon compared to the ELM-AFGST. The proposed controller can be seen to have reduced the system’s jitter to a large extent. It shows that the RLS-ELM network designed can estimate the unknown disturbances of the system while making online adjustments to suppress the jitter caused by nonlinearities and uncertainties, which ensures the fixed-time stability of the system while avoiding the generation of excessive estimates when the disturbances vary greatly, and thus obtains more excellent stability. These results highlight the effectiveness of the ELM-AFGST controller when it comes to stable tracking performance during start-up and transition, and therefore, the ELM-AFGST controller designed is suitable for practical unmanned agricultural machine travel.

#### 4.1.3 Comparison of the effects of different observers

**Figure 11:**
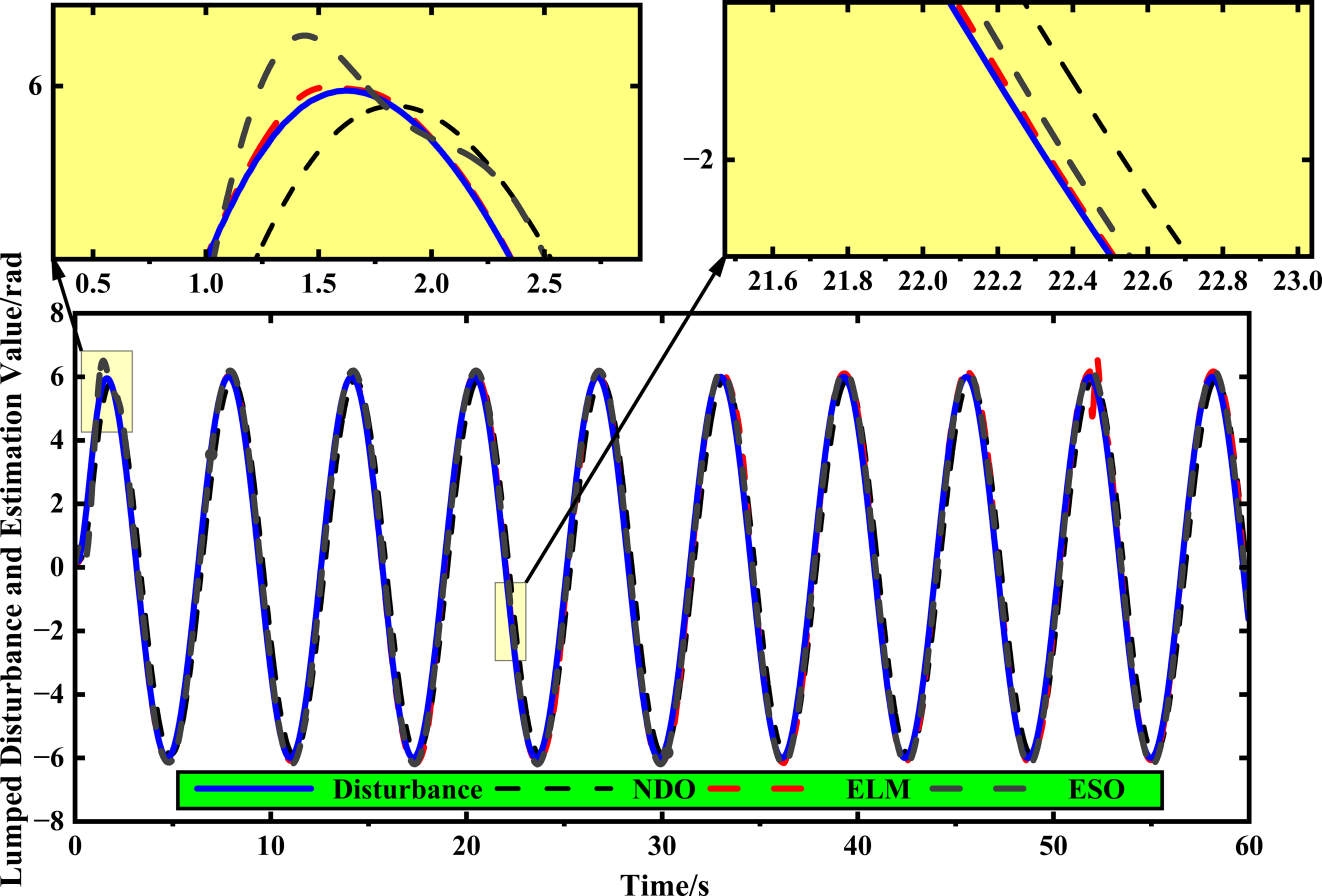
The lumped disturbance estimation under RLS-ELM, ESO, and NDO.

The above simulation comparison results in two different cases show that ELM-AFGST achieves better path control performance compared to other control methods. However, to better reflect the advantages of the RLS-ELM disturbance estimation designed, it needs to be verified in comparison with other common NDO and ESO observers. The results of disturbance estimation comparison are shown in **Figure 11**. From the figure, it can be seen that RLS-ELM provides a more accurate estimation of the disturbance when the disturbance signal changes sharply in the initial stage and does not require an accurate upper bound value of the disturbance, and compared with the rest of the observation compensators, it has the best estimation of the disturbances. It is better suitable to agricultural terrain scenarios.

**Figure 12:**
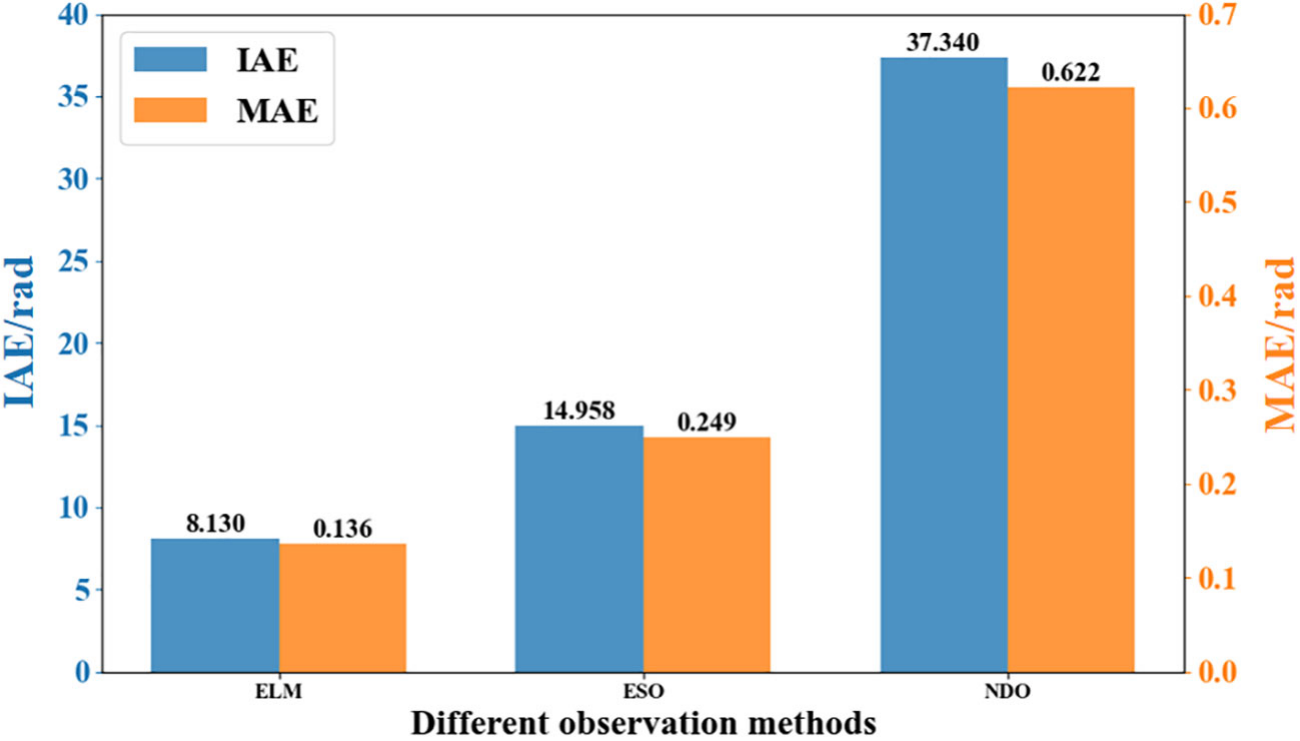
Comparison of observational performance with different metrics.

Considering that the disturbance observation error is an important index to measure the performance of the observer ultimately, the integral absolute error (IAE) and the mean absolute error (MAE) are used as the performance indexes, which are shown in **Figure 12** for different compensation controls. From the comparison results in the figure, it is clear that the observation accuracy of RLS-ELM is better than that of the other observers. It is worth noting that the state observation in path tracking control systems plays a crucial role in solving practical engineering problems. Not all state variables can be directly measured during the travelling process of the tracked harvester. The system compensation by RLS-ELM can provide complete and accurate perturbation state information for the controller designed, thus improving the tracking accuracy.

### 4.2 Analysis of site experiment results

**Figure 13:**
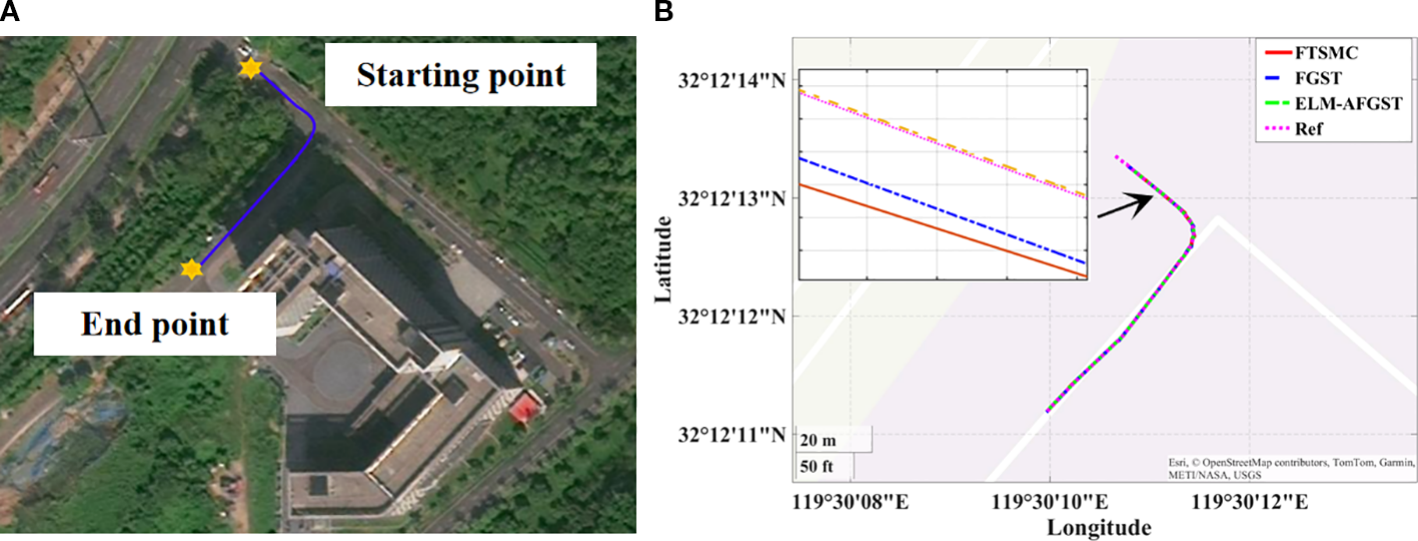
Actual scenarios. **(A)** satellite map trajectory map **(B)** path trajectories under different control schemes.

To verify the feasibility of the method proposed in this research, a comparative experiment under different path tracking algorithms was carried out through the reference path on the map, the experiment time was May 2025, the location is in the vicinity of Leisi Building, Jiangsu University, Zhenjiang City, Jiangsu Province, China the desired path is shown in **Figure 13**. The concrete ground is hard, and the overall road surface condition is good. However, the presence of surface unevenness or stones on the road surface has a certain impact on the path control effect, which can be considered as a form of matching disturbance. Analyzing the deviation results between the actual driving path and the desired path is an important indicator of control performance. The actual driving environment is more complex compared to the computer simulation; the situation encountered is more complicated, which will further enhance the difficulty of controlling the combine harvester. Therefore, to avoid the error effects generated by the jitter error when the harvester starts to move forward, the tracking operation is carried out after the harvester has travelled smoothly and straightly for three seconds, and the position data is recorded in real time. The vehicle’s lateral error under the initial state is 2 m, and the heading error is 0 rad.

**Figure 14:**
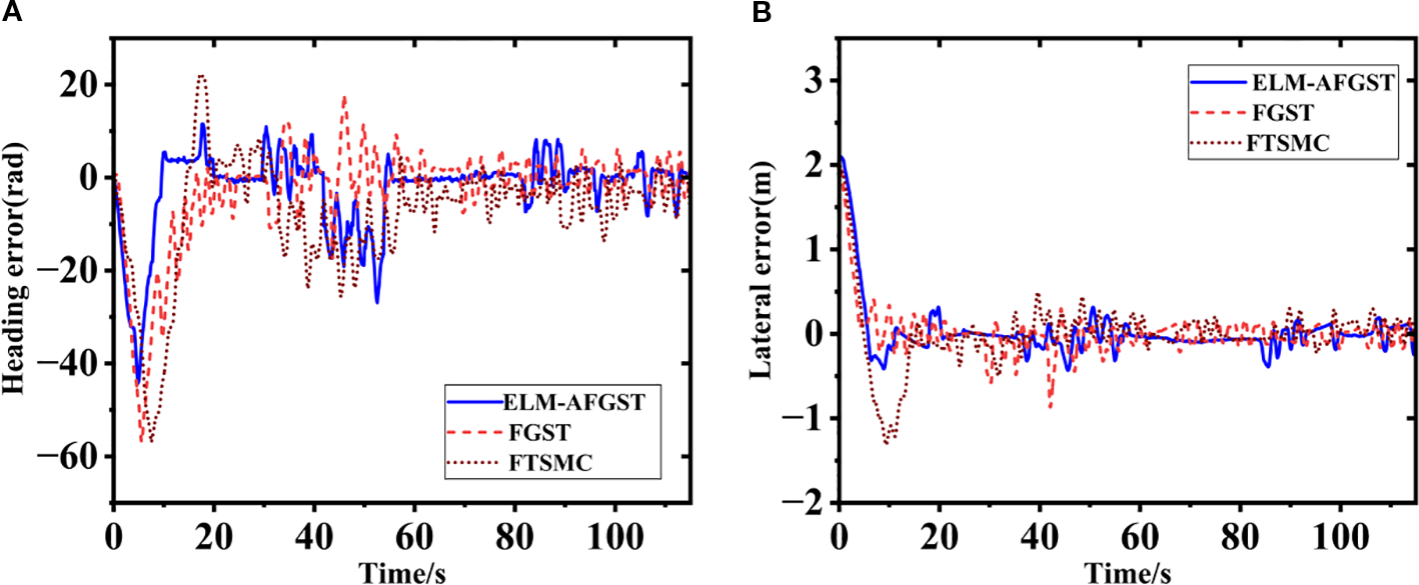
Actual path tracking results **(A)** lateral error; **(B)** heading error.

The error during path tracking under different control schemes are shown in **Figure 14**. From the error results, the proposed control scheme has a better control effect in eliminating the path error, and compared with the other control scheme, the ELM-AFGST control scheme has the fastest convergence in tracking error and the smallest amount of overshooting. In the simulation experiment, the convergence time was 4.68 s, while in the actual test process, the convergence time was 8.84 s. The main reason for this is that in actual testing, to avoid overloading of the mechanical structure due to rapid steering, the hydraulic controller embedded dynamic constraints (steering angular velocity not exceeding 0.5 rad/s), which were not set in the simulation. Although this engineering design reduces the risk of mechanical wear and tear, it objectively delays the convergence speed of errors, resulting in actual convergence time being longer than the simulation results and when the hydraulic pressure switches direction (such as adjusting from left to right), it is necessary to first overcome the empty stroke caused by mechanical clearance, which is ignored in the simulation and increases additional adjustment time. And the calculation delay of GNSS positioning module will also increase the time, so the final convergence time is greater than the actual simulation time.

**Figure 15:**
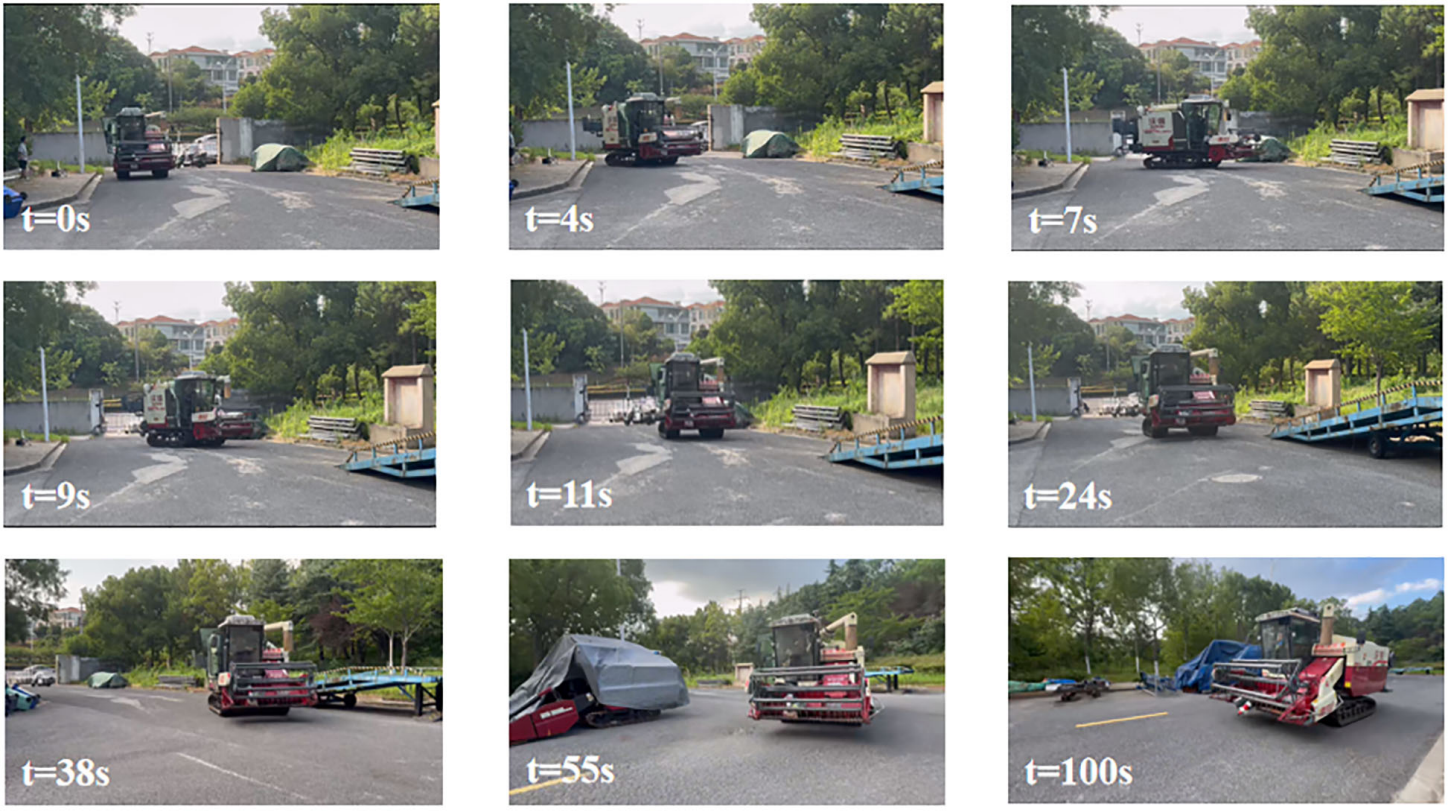
Combine harvester path tracking process.

The experiment results show that the harvester’s tracking accuracy in turns is lower than during straight-line navigation. This is mainly because, during a curve, the combine harvester’s angular velocity varies frequently, and there is a certain lag in the steering actuator. When driving in a curve, the actuator lags in its response time, leading to increased tracking errors. Throughout the entire tracking process, to better demonstrate the superiority of the proposed scheme, the MAE is used in **Table 3** to represent lateral and heading errors across different control schemes. The scheme introduced in this study has the best control performance. Specifically, compared to the FGST and FTSMC control schemes, the average lateral errors with ELM-AFGST control scheme decreases by approximately 24.5% and 27.4%, respectively. Regarding heading deviation, the average error is reduced by about 5.4% and 30.8% compared to the FGST and FTSMC schemes, respectively, and no out-of-control incidents occur during operation, the path tracking process is shown in **Figure 15**.

**Table 3:** Comparison of performance indicators of different control schemes.

Control scheme	Lateral error		Heading error	
	RMSE	MAE	RMSE	MAE
FTSMC	0.1492	0.1142	0.3910	0.2130
FGST	0.1453	0.1097	0.3228	0.1558
ELM-AFGST	0.1159	0.0828	0.2756	0.1473

The experiment results demonstrate that the designed control scheme exhibits good control performance in the actual driving environment and does not cause the system to crash due to computational burden, thus ensuring the standard recording of experiment data, in the vehicle tracking scene, adjusting the vehicle speed has limited impact on the overall tracking effect. The tracking algorithm can adaptively adjust through the state update equation to maintain a stable estimation of the target position and trajectory, so there is no need to pay too much attention to the impact of vehicle speed fine-tuning on the tracking results. In the complex field environment, the harvester driving performance will be affected by many factors, such as the relative unevenness of the road surface or the presence of weeds and clods of soil, the attitude of the entire machine is more undulating when it is travelling, which leads to an increase in the amount of offset, and therefore the offset distance is greater than that during the experiment conducted in the simulation environment.

## 5 Discussion

The proposed ELM-AFGST path-tracking controller has demonstrated strong performance in both simulations and experiments, confirming its ability to handle external disturbances and maintain stability. However, a more detailed analysis highlights several key considerations: Compared with traditional path tracking control methods FTSMC and FGST, the proposed ELM-AFGST method achieves superior robustness and accuracy. The design of a generalized terminal sliding mode surface with adaptive gain coefficients improves adaptability, while the integration of RLS-ELM provides online learning to mitigate uncertain disturbances. These contributions address the shortcomings of conventional controllers, which often suffer from sensitivity to parameter tuning and degraded performance under variable disturbances. The proposed controller therefore narrows an important gap by combining fixed-time convergence with adaptive intelligence, offering both stability and flexibility in dynamic agricultural environments. This study verifies the effectiveness of the proposed control strategy based on a kinematic model. Although the design process of path tracking control is simplified, it does not fully describe the key track slip characteristics in unstructured agricultural environments, which directly leads to deviations between the actual driving trajectory and the theoretical path. Meanwhile, the model ignores the dynamic coupling effect of the load, which in turn affects the steering response and driving stability. These dynamic deviations have not been included in the current control system.

This control method has been validated to meet the operational requirements of autonomous agricultural vehicles in structured road environments. However, its potential application in unstructured field scenarios has not yet been tested. Future work will be focused on testing in an unstructured environment, under conditions such as variable soil moisture contents (simulating soft fields), residual crop coverage, and cross-slope driving to evaluate the control effect on lateral errors and heading deviations caused by track slippage. Load variations (e.g. empty-load and fully-load conditions) should also be considered to assess adaptability under dynamic working states and determine whether the existing model can cope with the dynamic disturbances unique to unstructured environments. Moreover, in the field of plant protection, the autonomous sprayers equipped with the path tracking technology can accurately drive along the crop lines or the pest areas identified by sensors, reducing the risk of environmental pollution while improving effectiveness. Similarly, in pest and disease monitoring, autonomous inspection equipment combined with path-tracking technology can collect data according to the preset path, ensuring full coverage of key monitoring points in complex terrain, helping to detect early signs of pest and disease outbreaks, and supporting precise prevention and control.

While the proposed controller improves adaptability, further work is needed to enhance robustness in highly dynamic conditions. Current parameter tuning and ELM node selection are still empirical, and fixed parameters are difficult to adapt to dynamic disturbances in the field. In the future, dynamic model constraints need to be introduced, and the integration of parameter adaptive optimization algorithms such as reinforcement learning with existing control frameworks needs to be explored. By dynamically adjusting the weights of core parameters online, the dependence on manual experience can be reduced, and the system can maintain stable control performance under different crop types, soil conditions, and operating modes, further expanding the applicability of the technology.

## 6 Conclusion

This study addressed the challenge of path tracking for autonomous navigation of tracked harvesters under external disturbance conditions. An AFGST control method integrated with RLS-ELM is developed, and its effectiveness is verified through simulation and structured road tests. The research results show that the proposed controller achieves fixed-time and significantly reduces tracking errors compared with conventional controllers, lowering lateral error by about 24-27% and heading deviation by 5-31%. These findings confirm that the method provides robust and precise path-tracking performance, meeting the requirements for unmanned operation of agricultural vehicles. The practical contribution lies in offering a reliable control strategy that supports high-precision autonomous navigation, thereby improving operational efficiency and providing technical support for promoting the transformation of agricultural production towards intelligence and sustainability. Future research will extend validation to unstructured field environments and incorporate dynamic modeling and adaptive parameter optimization to further improve system robustness and adaptability in complex agricultural scenarios.

## Data Availability

The original contributions presented in the study are included in the article/supplementary material. Further inquiries can be directed to the corresponding author.

## References

[B1] Bai Y. Zhang B. Xu N. Zhou J. Shi J. Diao Z. (2023). Vision-based navigation and guidance for agricultural autonomous vehicles and robots: A review. Comput. Electron. Agric. 205, 107584. doi: 10.1016/j.compag.2022.107584

[B2] Carpio R. F. Potena C. Maiolini J. Ulivi G. Rosselló N. B. Garone E.. (2020). A navigation architecture for ackermann vehicles in precision farming. IEEE Robotics Automation Lett. 5, 1103–1110. doi: 10.1109/LSP.2016.

[B3] Chen T. Xu L. Ahn H. S. Lu E. Liu Y. Xu R. (2023). Evaluation of headland turning types of adjacent parallel paths for combine harvesters. Biosyst. Eng. 233, 93–113. doi: 10.1016/j.biosystemseng.2023.07.009

[B4] Cheng Z. Lu Z. (2018). Research on the PID control of the ESP system of tractor based on improved AFSA and improved SA. Comput. Electron. Agric. 148, 142–147. doi: 10.1016/j.compag.2018.03.013

[B5] Cui B. Cui X. Wei X. Zhu Y. Ma Z. Zhao Y.. (2024). Design and testing of a tractor automatic navigation system based on dynamic path search and a fuzzy stanley model. Agriculture 14, 2136. doi: 10.3390/agriculture14122136

[B6] Ding C. Ding S. Wei X. Ji X. Sun J. Mei K. (2023). Disturbance-observer-based barrier function adaptive sliding mode control for path tracking of autonomous agricultural vehicles with matched-mismatched disturbances. IEEE Trans. Transportation Electrification 10, 6748–6760. doi: 10.1109/TTE.2023.3333001

[B7] Ding C. Ding S. Wei X. Mei K. (2022). Output feedback sliding mode control for path-tracking of autonomous agricultural vehicles. Nonlinear Dynamics 110, 2429–2445. doi: 10.1007/s11071-022-07739-2

[B8] Ding S. Huang C. Ding C. Wei X. (2023). Straight-line tracking controller design of agricultural tractors based on third-order sliding mode. Comput. Electrical Eng. 106, 108559. doi: 10.1016/j.compeleceng.2022.108559

[B9] Hu Q. Fan Z. Zhang X. Sun N. Li X. Qiu Q. (2025). Robust localization and tracking control of high-clearance robot system servicing high-throughput wheat phenotyping. Comput. Electron. Agric. 229, 109793. doi: 10.1016/j.compag.2024.109793

[B10] Ji X. Ding S. Cui B. Ding C. Wei X. (2023a). Barrier function-based nonsingular terminal sliding mode control for path tracking of tractor-like with experimental validation. IEEE Trans. Circuits Syst. II: Express Briefs 70, 3024–3028. doi: 10.1109/TCSII.2023.3248039

[B11] Ji X. Ding S. Wei X. Cui B. (2023b). Path tracking of unmanned agricultural tractors based on a novel adaptive second-order sliding mode control. J. Franklin Institute 360, 5811–5831. doi: 10.1016/j.jfranklin.2023.03.053

[B12] Ji X. Ding S. Wei X. Mei K. Cui B. Sun J. (2024). Path tracking control of unmanned agricultural tractors via modified supertwisting sliding mode and disturbance observer. IEEE/ASME Trans. Mechatronics. 29 (6), 4051–4062. doi: 10.1109/TMECH.2024.3360097

[B13] Ji X. Wei X. Wang A. Cui B. Song Q. (2022). A novel composite adaptive terminal sliding mode controller for farm vehicles lateral path tracking control. Nonlinear Dynamics 110, 2415–2428. doi: 10.1007/s11071-022-07730-x

[B14] Jin Y. Liu J. Xu Z. Yuan S. Li P. Wang J. (2021). Development status and trend of agricultural robot technology. Int. J. Agric. Biol. Eng. 14, 1–19. doi: 10.25165/j.ijabe.20211404.6821

[B15] Li Z. Chen L. Wang H. (2023). Fixed-time sliding mode-based adaptive path tracking control of maize plant protection robot via extreme learning machine. IEEE Robotics Automation Lett. 10 (7), 7396–7403. doi: 10.1109/LRA.2023.3244125

[B16] Luo Y. Wei L. Xu L. Zhang Q. Liu J. Cai Q.. (2022). Stereo-vision-based multi-crop harvesting edge detection for precise automatic steering of combine harvester. Biosyst. Eng. 215, 115–128. doi: 10.1016/j.biosystemseng.2021.12.016

[B17] Ma Z. Yin C. Du X. Zhao L. Lin L. Zhang G.. (2022). Rice row tracking control of crawler tractor based on the satellite and visual integrated navigation. Comput. Electron. Agric. 197, 106935. doi: 10.1016/j.compag.2022.106935

[B18] Moreno J. A. Osorio M. (2012). Strict Lyapunov functions for the super-twisting algorithm. IEEE Trans. automatic control 57, 1035–1040. doi: 10.1109/TAC.2012.2186179

[B19] Rovira-Más F. Saiz-Rubio V. Cuenca-Cuenca A. (2020). Augmented perception for agricultural robots navigation. IEEE Sensors J. 21, 11712–11727. doi: 10.1109/JSEN.2020.3016081

[B20] Si H. Li Y. Sun C. Qiao H. Hu X. (2019). A hierarchical game approach on real-time navigation scheduling of agricultural harvesters. Comput. Electron. Agric. 162, 112–118. doi: 10.1016/j.compag.2019.03.034

[B21] Sun J. Li Q. Ding S. Xing G. Chen L. (2023). Fixed-time generalized super-twisting control for path tracking of autonomous agricultural vehicles considering wheel slipping. Comput. Electron. Agric. 213, 108231. doi: 10.1016/j.compag.2023.108231

[B22] Sun J. Wang Z. Ding S. Xia J. Xing G. (2024). Adaptive disturbance observer-based fixed time nonsingular terminal sliding mode control for path-tracking of unmanned agricultural tractors. Biosyst. Eng. 246, 96–109. doi: 10.1016/j.biosystemseng.2024.06.013

[B23] Wang B. Du X. Wang Y. Mao H. (2024). Multi-machine collaboration realization conditions and precise and efficient production mode of intelligent agricultural machinery. Int. J. Agric. Biol. Eng. 17, 27–36. doi: 10.25165/j.ijabe.20241702.8127

[B24] Xie B. Jin Y. Faheem M. Gao W. Liu J. Jiang H.. (2023). Research progress of autonomous navigation technology for multi-agricultural scenes. Comput. Electron. Agric. 211, 107963. doi: 10.1016/j.compag.2023.107963

[B25] Xu G. Chen M. He X. Pang H. Miao H. Cui P.. (2021). Path following control of tractor with an electro-hydraulic coupling steering system: Layered multi-loop robust control architecture. Biosyst. Eng. 209, 282–299. doi: 10.1016/j.biosystemseng.2021.07.014

[B26] Yang W. Ding S. Ding C. (2024). Fast supertwisting sliding mode control with antipeaking extended state observer for path-tracking of unmanned agricultural vehicles. IEEE Trans. Ind. Electron. 71, 12973–12982. doi: 10.1109/TIE.2024.3355507

[B27] Yang Y. Li Y. Wen X. Zhang G. Ma Q. Cheng S.. (2022). An optimal goal point determination algorithm for automatic navigation of agricultural machinery: Improving the tracking accuracy of the Pure Pursuit algorithm. Comput. Electron. Agric. 194, 106760. doi: 10.1016/j.compag.2022.106760

[B28] Zhang T. Jiao X. Lin Z. (2022). Finite time trajectory tracking control of autonomous agricultural tractor integrated nonsingular fast terminal sliding mode and disturbance observer. Biosyst. Eng. 219, 153–164. doi: 10.1016/j.biosystemseng.2022.04.020

[B29] Zhang Y. Shen Y. Liu H. He S. Khan Z. (2025). A composite sliding mode controller with extended disturbance observer for 4WSS agricultural robots in unstructured farmlands. Comput. Electron. Agric. 232, 110069. doi: 10.1016/j.compag.2025.110069

[B30] Zhang Y. Zhang B. Shen C. Liu H. Huang J. Tian K.. (2024). Review of the field environmental sensing methods based on multi-sensor information fusion technology. Int. J. Agric. Biol. Eng. 17, 1–13. doi: 10.25165/j.ijabe.20241702.8596

[B31] Zou W. Xia Y. Cao W. (2022). Back-propagation extreme learning machine. Soft Computing 26, 9179–9188. doi: 10.1007/s00500-022-07331-1

[B32] Zuo Z. (2015). Non-singular fixed-time terminal sliding mode control of non-linear systems. IET control Theory Appl. 9, 545–552. doi: 10.1049/iet-cta.2014.0202

